# EVM005: An Ectromelia-Encoded Protein with Dual Roles in NF-κB Inhibition and Virulence

**DOI:** 10.1371/journal.ppat.1004326

**Published:** 2014-08-14

**Authors:** Nicholas van Buuren, Kristin Burles, Jill Schriewer, Ninad Mehta, Scott Parker, R. Mark Buller, Michele Barry

**Affiliations:** 1 Li Ka Shing Institute of Virology, Department of Medical Microbiology and Immunology, University of Alberta, Edmonton, Alberta, Canada; 2 Department of Molecular Microbiology and Immunology, Saint Louis University School of Medicine, St. Louis, Missouri, United States of America; University of Florida, United States of America

## Abstract

Poxviruses contain large dsDNA genomes encoding numerous open reading frames that manipulate cellular signalling pathways and interfere with the host immune response. The NF-κB signalling cascade is an important mediator of innate immunity and inflammation, and is tightly regulated by ubiquitination at several key points. A critical step in NF-κB activation is the ubiquitination and degradation of the inhibitor of kappaB (IκBα), by the cellular SCF^β-TRCP^ ubiquitin ligase complex. We show here that upon stimulation with TNFα or IL-1β, *Orthopoxvirus*-infected cells displayed an accumulation of phosphorylated IκBα, indicating that NF-κB activation was inhibited during poxvirus infection. Ectromelia virus is the causative agent of lethal mousepox, a natural disease that is fatal in mice. Previously, we identified a family of four ectromelia virus genes (EVM002, EVM005, EVM154 and EVM165) that contain N-terminal ankyrin repeats and C-terminal F-box domains that interact with the cellular SCF ubiquitin ligase complex. Since degradation of IκBα is catalyzed by the SCF^β-TRCP^ ubiquitin ligase, we investigated the role of the ectromelia virus ankyrin/F-box protein, EVM005, in the regulation of NF-κB. Expression of Flag-EVM005 inhibited both TNFα- and IL-1β-stimulated IκBα degradation and p65 nuclear translocation. Inhibition of the NF-κB pathway by EVM005 was dependent on the F-box domain, and interaction with the SCF complex. Additionally, ectromelia virus devoid of EVM005 was shown to inhibit NF-κB activation, despite lacking the EVM005 open reading frame. Finally, ectromelia virus devoid of EVM005 was attenuated in both A/NCR and C57BL/6 mouse models, indicating that EVM005 is required for virulence and immune regulation *in vivo*.

## Author Summary

Poxviruses are large dsDNA viruses that are renowned for regulating cellular pathways and manipulating the host immune response, including the NF-κB pathway. NF-κB inhibition by poxviruses is a growing area of interest and this family of viruses has developed multiple mechanisms to manipulate the pathway. Here, we focus on regulation of the NF-κB pathway by ectromelia virus, the causative agent of mousepox. We demonstrate that ectromelia virus is a potent inhibitor of the NF-κB pathway. Previously, we identified a family of four ectromelia virus genes that contain N-terminal ankyrin repeats and a C-terminal F-box domain that interacts with the cellular SCF ubiquitin ligase. Significantly, expression of the ankyrin/F-box protein, EVM005, inhibited NF-κB, and the F-box domain was critical for NF-κB inhibition and interaction with the SCF complex. Ectromelia virus devoid of EVM005 still inhibited NF-κB, indicating that multiple gene products contribute to NF-κB inhibition. Importantly, mice infected with ectromelia virus lacking EVM005 had a robust immune response, leading to viral clearance during infection. The data present two mechanisms, one in which EVM005 inhibits NF-κB activation through manipulation of the host SCF ubiquitin ligase complex, and an additional, NF-κB-independent mechanism that drives virulence.

## Introduction

The NF-κB family of transcription factors activate potent pro-inflammatory and anti-viral immune responses that are activated by a variety of signalling pathways [1], [2]. The family consists of five members, p50, p52, p65 (RelA), RelB, and c-Rel, which function as homo- or heterodimers to activate specific genes. The best-characterized NF-κB dimer is the p50/p65 heterodimer, which is held inactive in the cytoplasm by the inhibitor of κB (IκBα) [1], [2]. Signalling cascades initiated by both tumour necrosis factor α (TNFα) and interleukin 1β (IL-1β) trigger the activation of a set of kinases known as the IκB kinase (IKK) complex, which is composed of IKKα, IKKβ and IKKγ/NF-κB essential modifier (NEMO) [2]. Upon activation of the IKK complex, IKKβ phosphorylates IκBα on serines 32 and 36, targeting IκBα for polyubiquitination and degradation by the 26S proteasome [1], [2]. The SCF (Skp1/Cul1/F-box) ubiquitin ligase recruits phospho-IκBα through the F-box domain-containing adaptor protein, β-TRCP, resulting in the degradation of IκBα, and translocation of the p50/p65 heterodimer into the nucleus [1], [2].

Regulation of NF-κB signalling is common amongst most viruses, with each virus employing a combination of specifically tailored strategies [3]–[5]. For example, human immunodeficiency virus (HIV), human T-lymphotropic virus type 1 (HTLV-1), hepatitis B virus (HBV), and Epstein-Barr virus (EBV) activate the NF-κB signalling pathway [4]. Virus activation of the NF-κB pathway could serve several roles. For instance, viruses that lack anti-apoptotic mechanisms may activate NF-κB to prolong the life of the infected cell in order to complete the viral replication cycle. In the case of EBV, constitutive activation of NF-κB leads to the up-regulation of NF-κB-regulated pro-survival proteins during latency [6]. Alternatively, HIV-1 contains NF-κB binding sites in the long terminal repeat (LTR) region of the genome that mediate HIV-1 gene expression [7]. In contrast, other viruses encode proteins that specifically inhibit NF-κB signalling [3]–[5]. For example, the V and C proteins encoded by the *Paramyxoviridae* associate with the STAT family of transcription factors, thus inhibiting the interferon response and NF-κB activation [8]. Moreover, African Swine Fever Virus encodes a homolog to IκBα that sequesters p65 in the cytoplasm following IκBα degradation [9]. Overall, the varieties of viral proteins that manipulate NF-κB indicate the importance of the long and varied relationship with NF-κB.

The inhibition of NF-κB by poxviruses has become a growing area of interest [5]. The *Poxviridae* is composed of viruses possessing large dsDNA genomes, encoding between 150 to 300 open reading frames [10]. Poxviruses are unique amongst DNA viruses in that they replicate in the cytoplasm, within DNA-rich regions termed “virus factories” [10]. Members of the *Orthopoxvirus* genus are well studied, and include variola virus, vaccinia virus (VACV), monkeypox virus, cowpox virus (CPXV), and the mouse-specific pathogen, ectromelia virus (ECTV) [11]. Poxviruses are renowned for the plethora of immune evasion mechanisms they encode; including mechanisms that regulate NF-κB [5], [12], [13]. One of the first identified mediators of NF-κB activation was M-T2, a secreted soluble virus version of the tumor necrosis factor receptor (vTNFR) [14], [15]. Soluble vTNFRs and vIL-1Rs were subsequently identified in a variety of poxviruses [13]. More recently, focus has shifted to the identification of intracellular inhibitors of NF-κB encoded by poxviruses [5]. VACV encodes three proteins, K7, A46, and A52, which contain Toll/IL-1 receptor (TIR) cytoplasmic domains and disrupt NF-κB activation triggered through the IL-1/Toll receptor pathway [16]–[18]. Additionally, the VACV-encoded proteins, B14, M2, K1, A49, and N1, disrupt NF-κB activation triggered through the TNFR pathway [19]–[22]. These proteins function at different points in the signalling cascade, clearly highlighting the importance of NF-κB inhibition during infection [19]–[24].

Recently, we identified a family of four ankyrin/F-box proteins encoded by ECTV, EVM002, EVM005, EVM154 (recently renamed EVM159), and EVM165 (recently renamed EVM170) that interact with the cellular SCF ubiquitin ligase complex [25]. The ECTV-encoded proteins contain N-terminal ankyrin repeats in conjunction with a C-terminal F-box domain; similar viral ankyrin/F-box proteins have been found in a wide range of poxviruses [26]. To date, no cellular F-box proteins have been found in conjunction with ankyrin repeats, suggesting that poxviruses, including ECTV, have evolved a novel set of genes to regulate the cellular SCF ubiquitin ligase. Multiple orthologs for EVM002, EVM154, and EVM165 exist within the poxvirus family; however, EVM005 has only one ortholog, CPXV-BR011, in CPXV virus strain Brighton Red, suggesting that EVM005 and CPXV-BR011 may play an important role that is specific to ECTV and CPXV. Since degradation of IκBα is catalyzed by the SCF^β-TRCP^ ubiquitin ligase, we investigated the role of EVM005 in regulation of IκBα degradation. Here, we show that cells infected with ECTV and stimulated with TNFα or IL-1β accumulate phosphorylated IκBα, indicating that IκBα is stabilized and not degraded. Ectopic expression of Flag-EVM005 inhibited both TNFα- and IL-1β-stimulated IκBα degradation and subsequent nuclear translocation of NF-κB; however, deletion of the EVM005 F-box domain resulted in activation of NF-κB. ECTV devoid of EVM005, ECTV-Δ005, inhibited NF-κB activation. Finally, we demonstrated that EVM005 is a critical virulence factor, since ECTV-Δ005 was attenuated in both A/NCR and C57BL/6 mice compared to wild type ECTV. These data demonstrate a novel role for poxvirus-encoded ankyrin/F-box proteins in regulation of the SCF ubiquitin ligase and NF-κB signalling.

## Results

### Ectromelia virus infection blocks IκBα degradation

**Figure 1:**
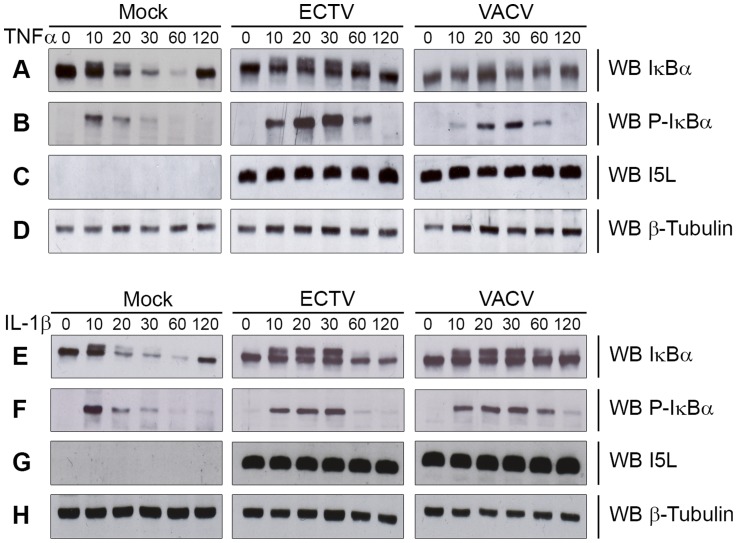
ECTV infection inhibits IκBα degradation. HeLa cells were mock-infected or infected with ECTV or VACV at a MOI of 5 for 12 hours and stimulated with 10 ng hours and stimulated with 10 ng ng/ml TNFα (A–D) or 10 ng ng/ml IL-1β (E–H). Protein samples were collected at 0, 10, 20, 30, 60 and 120 minutes post treatment. Cellular lysates were western blotted with anti minutes post treatment. Cellular lysates were western blotted with anti-IκBα (A and E), anti-phospho-IκBα (B and F), anti-I5L (C and G), and anti-β-tubulin (D and H).

The NF-κB signalling cascade activates a family of transcription factors responsible for initiating the pro-inflammatory response and antiviral innate immunity [1], [2]. Recent evidence indicates that many poxviruses encode proteins that tightly regulate the activation of NF-κB through the expression of secreted and intracellular factors [5], [12]. Unlike strains of VACV, ECTV lacks M2, K7, B14, A49 and A52, all of which are important inhibitors of NF-κB activation [16], [18], [21], [23], [24]. Given the absence of these inhibitors, we sought to determine if ECTV infection inhibited NF-κB activation. Since the degradation of IκBα is crucial for activation of the NF-κB pathway, we examined the kinetics of IκBα degradation during infection. HeLa cells were mock-infected, or infected with ECTV or VACV for 12 hours and treated with TNFα for up to 120 minutes. Mock-infected cells treated with TNFα showed a typical pattern of IκBα degradation kinetics (Fig. 1A). As early as 10 minutes post-TNFα treatment, mock-infected cells showed phosphorylated IκBα, as indicated by a doublet, which was subsequently degraded (Fig. 1A and B) [27], [28]. ECTV- and VACV-infected cells treated with TNFα also showed obvious phosphorylation of IκBα (Fig. 1A and B); however, the levels of both IκBα and phospho-IκBα were sustained compared to mock-infected cells (Fig. 1A and B). Interestingly, we did observe that lower levels of phospho-IκBα accumulated in cells infected with VACV compared to ECTV, and accumulation was delayed in comparison to ECTV-infected cells (Fig. 1A). Western blotting for I5L, a late poxviral protein, and cellular β-tubulin were used as loading controls (Fig. 1C and D) [25]. Similar observations were also seen following treatment with IL-1β (Fig. 1E–H), indicating that members of *Orthopoxvirus* genera, including ECTV, sustained levels of phospho-IκBα and inhibited the degradation of IκBα.

**Figure 2:**
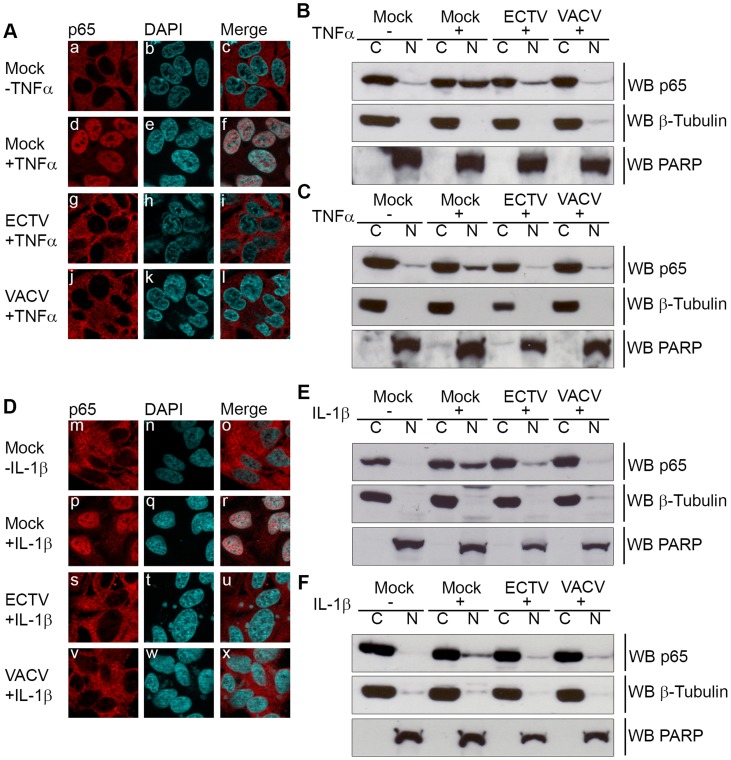
ECTV infected cells inhibit p65 nuclear translocation. (A and D) HeLa cells were mock-infected or infected with ECTV or VACV at a MOI of 5 for 12 hours followed by stimulation with 10 ng hours followed by stimulation with 10 ng ng/ml TNFα (A) or 10 ng ng/ml IL-1β (D) for 20 minutes. Cells were stained with anti minutes. Cells were stained with anti-p65 and DAPI to visualize nuclei and viral factories. (B and E) HeLa cells were mock-infected or infected with ECTV or VACV for 12 hours followed by stimulation with 10 ng hours followed by stimulation with 10 ng ng/ml TNFα (B) or 10 ng ng/ml IL-1β (E) for 20 minutes. Nuclear and cytoplasmic extracts were collected and western blotted with anti minutes. Nuclear and cytoplasmic extracts were collected and western blotted with anti-p65, anti-β-tubulin, and anti-PARP. (C and F) MEF cells were mock-infected or infected with ECTV or VACV for 12 hours followed by stimulation with 10 ng hours followed by stimulation with 10 ng ng/ml TNFα (C) or 10 ng ng/ml IL-1β (F) for 20 minutes. Nuclear and cytoplasmic extracts were collected and western blotted with anti minutes. Nuclear and cytoplasmic extracts were collected and western blotted with anti-p65, anti-β-tubulin, and anti-PARP.

Since IκBα appeared to be phosphorylated but not rapidly degraded during infection with ECTV or VACV, we sought to determine if NF-κB p65 was retained within the cytoplasm. HeLa cells were mock-infected or infected with ECTV or VACV, and p65 nuclear accumulation was assayed using immunofluorescence (Fig. 2A and D). Mock-infected cells demonstrated cytoplasmic retention of p65 in the absence of TNFα or IL-1β stimulation, as expected (Fig. 2A panels a–c and D panels m–o). In contrast, mock-infected cells stimulated with TNFα or IL-1β showed nuclear accumulation of p65 (Fig. 2A panels d–f and D panels p–r). Upon infection with ECTV or VACV, p65 was retained in the cytoplasm following treatment with TNFα or IL-1β, indicating that ECTV could inhibit NF-κB despite the lack of orthologs of M2, K7, B14, A49, and A52 (Fig. 2A panels g–l and D panels s–x). These data were confirmed by Western blotting cytoplasmic and nuclear extracts from infected HeLa cells with an antibody specific for p65 (Fig. 2B and E). As expected, p65 was absent from the nuclear extract of mock-infected cells. In contrast, mock-infected cells treated with TNFα or IL-1β showed nuclear p65 (Fig. 2B and E). Little p65 accumulation in the nuclear extract was observed in cells infected with ECTV or VACV and treated with TNFα or IL-1β (Fig. 2B and E). These results were also confirmed in mouse embryonic fibroblasts (MEF) (Fig. 2C and F). Together, these data indicate that NF-κB signalling is inhibited upon infection with members of the *Orthopoxvirus* genus. Importantly, despite the lack M2, K7, B14, A49 and A52 in ECTV, ECTV infection clearly inhibited p65 translocation to the nucleus.

### ECTV-encoded ankyrin/F-box protein, EVM005, inhibits p65 nuclear accumulation

Recently, we identified a family of four ankyrin/F-box proteins in ECTV (EVM002, EVM005, EVM154 and EVM165), which interact with the cellular SCF ubiquitin ligase (Fig. S1) [25]. The poxvirus family of ankyrin/F-box proteins differs substantially from the cellular F-box proteins. In contrast to the cellular F-box proteins, the poxviral F-box domains are found at the C-terminus in combination with N-terminal ankyrin repeats [25], [26], [29]–[34]. With the exception of EVM005, which has only one ortholog in cowpox virus Brighton Red, CPXVBR011, multiple orthologs exist for EVM002, EVM154 and EVM165. Since the SCF^β-TRCP^ ubiquitin ligase plays an essential role degrading phospho-IκBα, we sought to determine if EVM005 could inhibit IκBα degradation and NF-κB activation during ECTV infection [27], [28].

**Figure 3:**
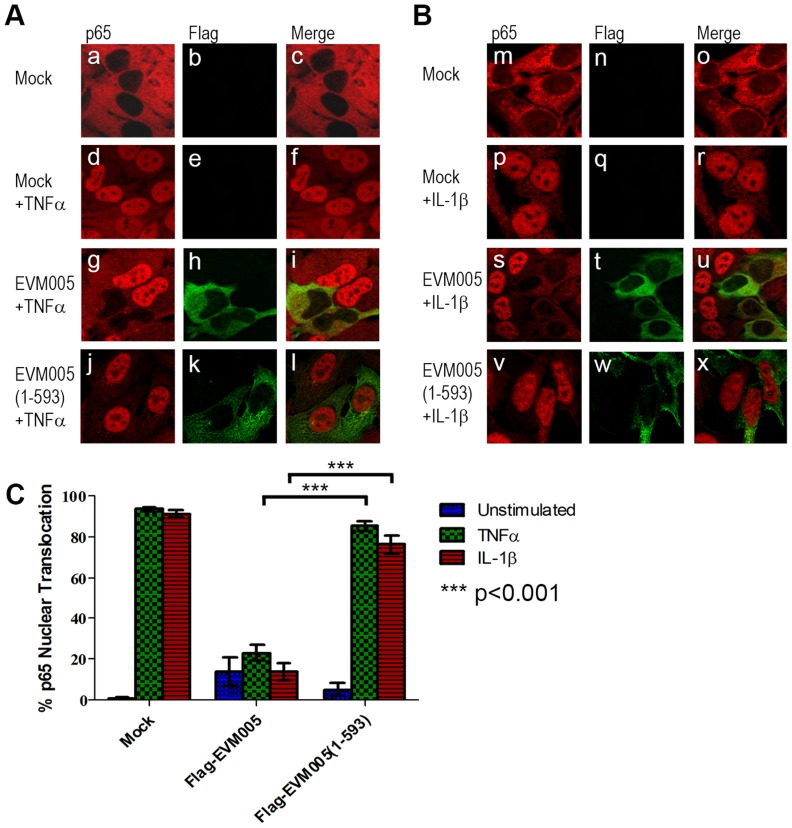
ECTV encoded EVM005 inhibits p65 nuclear accumulation. (A and B) HeLa cells were mock-transfected or transfected with pcDNA-Flag-EVM005 or pcDNA-Flag-EVM005(1-593). At 12 hours post transfection cells were treated with 10 ng hours post transfection cells were treated with 10 ng ng/ml TNFα (A) or 10 ng ng/ml IL-1β (B) for 20 minutes. Cells were fixed and stained with anti minutes. Cells were fixed and stained with anti-Flag to detect EVM005 and anti-p65. Cells were visualized by immunofluorescence. (C) Data was quantified by counting more than 50 cells in triplicate.

We first tested the ability of EVM005 to inhibit the nuclear accumulation of NF-κB p65. HeLa cells were mock-transfected or transfected with full length Flag-EVM005. At 12 hours post-transfection, cells were stimulated with TNFα or IL-1β for 20 minutes and nuclear accumulation of p65 was detected using immunofluorescence (Fig. 3). As expected, unstimulated HeLa cells demonstrated cytoplasmic staining of p65 (Fig. 3A panels a–c and B panels m–o), and strong nuclear accumulation of p65 was seen following TNFα and IL-1β stimulation (Fig. 3A panels d–f and B panels p–r). In contrast, cells expressing Flag-EVM005 that were stimulated with TNFα or IL-1β strongly inhibited p65 nuclear accumulation (Fig. 3A panels g–i and B panels s–u). Given the importance of the F-box domain in associating with the SCF ubiquitin ligase, we wanted to determine whether this domain also contributed to the inhibition of NF-κB activation. To do this, we utilized an EVM005 mutant, Flag-EVM005(1-593), which lacks the C-terminal F-box-like domain and fails to interact with the SCF ubiquitin ligase [25]. Interestingly, cells expressing Flag-EVM005(1-593) displayed strong nuclear staining of p65 following TNFα or IL-1β stimulation (Fig. 3A panels j–l and B panels v–x). Nuclear translocation of p65 was quantified by counting cells from three independent experiments (Fig. 3C). These data indicate that expression of Flag-EVM005 inhibited both TNFα- and IL-1β-induced nuclear accumulation of p65, and that inhibition of p65 nuclear accumulation required a functional F-box domain.

### EVM005 inhibits TNFα- and IL-1β-induced IκBα degradation

**Figure 4:**
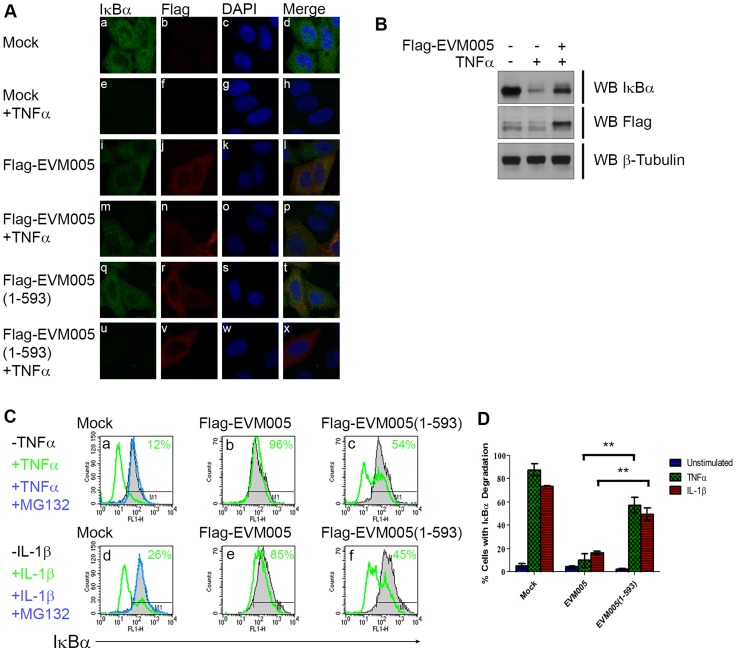
EVM005 inhibits IκBα degradation. (A) HeLa cells were mock-transfected or transfected with pcDNA-Flag-EVM005 or pcDNA-Flag-EVM005(1-593). At 12 hours post hours post-transfection, cells were untreated or treated with 10 ng ng/ml TNFα for 20 minutes. Cells were fixed and stained with DAPI, anti minutes. Cells were fixed and stained with DAPI, anti-IκBα, and anti-Flag, and visualized by confocal microscopy. (B) HeLa cells were mock-transfected or transfected with pcDNA-Flag-EVM005. At 12 hours post hours post-transfection, cells were left unstimulated or stimulated with 10 ng ng/ml TNFα for 20 min. Protein samples were separated by SDS min. Protein samples were separated by SDS-PAGE, and western blotted with anti-Flag, anti-IκBα, and anti-β-tubulin. (C) HeLa cells were mock-transfected or transfected with pcDNA-Flag-EVM005 or pcDNA-Flag-EVM005(1-593). Mock cells were untreated or pre-treated with 10 µM MG132 for one hour as a positive control for inhibition of IκBα degradation. At 18 hours post transfection, cells were stimulated with 10 ng hours post transfection, cells were stimulated with 10 ng ng/ml TNFα (panels a–c) or 10 ng ng/ml IL-1β (panels d–f) for 20 minutes. Cells were harvested, fixed and co minutes. Cells were harvested, fixed and co-stained with anti-Flag and anti-IκBα and samples were analyzed by flow cytometry. Flag-positive populations were gated on for analysis and IκBα fluorescence was measured on the x-axis. (D) Quantification of IκBα degradation was determined by measuring the percentage of cells with degraded IκBα. The experiment was performed in triplicate, and the standard error of the mean (SEM) was determined.

Since transient expression of EVM005 inhibited p65 translocation (Fig. 3), we sought to determine if EVM005 stabilized IκBα. HeLa cells were transfected with Flag-EVM005 and IκBα was visualized using immunofluorescence (Fig. 4A). As expected, in unstimulated cells IκBα was present within the cytoplasm (Fig. 4A panel a–d). Following 20 minutes of treatment with TNFα, the level of IκBα within the cytoplasm dramatically decreased as a result of ubiquitination and degradation of IκBα (Fig. 4A panel e–h) [27], [28]. Expression of Flag-EVM005 in the absence of TNFα stimulation showed that the levels of IκBα were unaffected (Fig. 4A panel i–l). In contrast, cells expressing Flag-EVM005 and stimulated with TNFα demonstrated that ectopic expression of EVM005 stabilized IκBα compared to the surrounding cells (Fig. 4A panel m–p). Levels of IκBα were unaffected by expression of Flag-EVM005(1-593) (Fig. 4A panel q–t); however, upon treatment with TNFα, IκBα was degraded, suggesting that the F-box domain was necessary for EVM005 to inhibit IκBα degradation (Fig. 4A panel u–x). To further confirm these data, HeLa cells were mock-transfected or transfected with Flag-EVM005 in the absence or presence of TNFα and immunoblotted for anti-IκBα, anti-Flag to detect EVM005, and anti-β-tubulin as a loading control. Compared to unstimulated cells, which showed a significant level of IκBα, cells treated with TNFα showed decreased levels of IκBα (Fig. 4B). Expression of EVM005 led to the stabilization of IκBα (Fig. 4B). Finally, we tested the ability of EVM005 to inhibit IκBα degradation by flow cytometry. HeLa cells were mock-transfected, or transfected with Flag-EVM005 or Flag-EVM005(1-593) [25]. Twenty-four hours post-transfection, cells were stimulated with TNFα or IL-1β, and fixed and stained with anti-Flag and anti-IκBα, to detect EVM005 and IκBα, respectively. Flag-positive cells were gated for analysis (Fig. 4C panels b and e). Untransfected cells demonstrated levels of IκBα that were significantly decreased following TNFα or IL-1β stimulation, as indicated by a leftward shift on the histogram (shown in green) (Fig. 4C panels a and d). Pre-treatment of HeLa cells with the proteasome inhibitor MG132, and subsequent TNFα or IL-1β stimulation (shown in blue) inhibited the degradation of IκBα, as expected (Fig. 4C panel a and d) [27]. HeLa cells expressing Flag-EVM005 and stimulated with TNFα or IL-1β inhibited IκBα degradation (Fig. 4C panels b and e); however, expression of Flag-EVM005(1-593) was unable to stabilize IκBα, resulting in degradation of IκBα (Fig. 4C panels c and f). This experiment was repeated in triplicate and these data were quantified by measuring the percentage of cells that underwent IκBα degradation (Fig. 4D). These data indicated that Flag-EVM005 strongly inhibits TNFα- and IL-1β-induced IκBα degradation, while the F-box deletion mutant failed to inhibit IκBα degradation (Fig. 4). Together, these data show that EVM005 expression blocked IκBα degradation in an F-box-dependent manner.

### ECTV-Δ005 is still a potent inhibitor of NF-κB activation

To further examine the role of EVM005 during infection, we generated an EVM005 deletion virus, ECTV-Δ005. In the past, deletion of open reading frames from poxvirus genomes has been performed by inserting drug selection or fluorescent markers into the gene of interest. Instead, we used the novel Selectable and Excisable Marker system that utilizes the Cre recombinase to delete the selection markers resulting a clean deletion of the targeted open reading frame [35], [36]. A cassette containing yellow fluorescent protein fused to guanine phosphoribosyl transferase *(yfp-gpt)* was inserted into the EVM005 locus. To generate a marker-free EVM005 deletion virus, ECTV-Δ005, we removed the *yfp-gpt* marker by infecting U20S cells that stably expressed a cytoplasmic mutant of the Cre recombinase (Fig. S2) [36]. Additionally, two revertant viruses were generated by replacing the *yfp-gpt* cassette with either wild type EVM005 or EVM005(1-593), a mutant lacking the F-box domain. PCR amplification of the EVM005 locus from viral genomes was used to screen for the purity of our viral products (Fig. S2). Using a multi-step growth curve, no growth defects were detected upon infection with ECTV, ECTV-Δ005, ECTV-005-rev or ECTV-005(1-593)-rev (Fig. S2).

**Figure 5:**
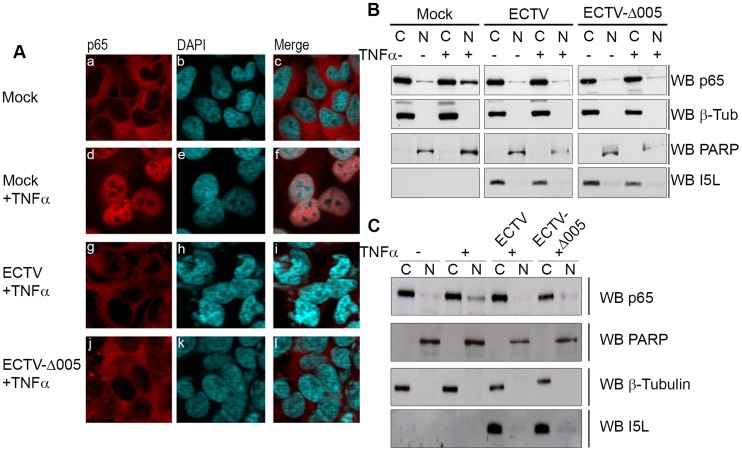
ECTV-Δ005 still inhibits p65 nuclear accumulation. (A) HeLa cells were mock-infected or infected with ECTV or ECTV-Δ005 at a MOI of 5. At 12 hours post infection, cells were stimulated with 10 ng hours post infection, cells were stimulated with 10 ng ng/ml TNFα for 20 minutes, fixed and stained with anti minutes, fixed and stained with anti-p65 and DAPI. Cells were visualized by immunofluorescence. (B) HeLa cells or (C) MEF cells were mock-infected or infected with ECTV or ECTV-Δ005 at a MOI of 5. At 12 hours post infection, cells were stimulated with 10 ng hours post infection, cells were stimulated with 10 ng ng/ml TNFα for 20 minutes, and nuclear and cytoplasmic extracts were collected. Protein samples were separated by SDS minutes, and nuclear and cytoplasmic extracts were collected. Protein samples were separated by SDS-PAGE and western blotted with anti-p65, anti-β-tubulin, anti-PARP and anti-I5L.

**Figure 6:**
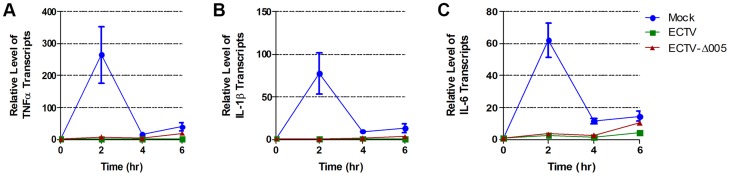
ECTV and ECTV-Δ005 inhibit production of NF-κB regulated transcripts. HeLa cells were mock-infected or infected with ECTV or ECTV-Δ005 at a MOI of 5. At 12 hours post infection cells were stimulated with TNF hours post infection cells were stimulated with TNFα. RNA samples were collected at the indicated time points post-TNFα treatment. Samples were reverse transcribed, followed by real time PCR analysis for relative levels of (A) TNFα, (B) IL-1β, and (C) IL-6 transcripts, as compared to GAPDH. Time courses were performed in triplicate and plotted as the average with standard error.

To determine if ECTV devoid of EVM005 could still inhibit nuclear accumulation of p65 following stimulation with TNFα, HeLa cells were mock-infected, or infected with ECTV or ECTV-Δ005. Immunofluorescence revealed that infection with ECTV-Δ005 inhibited NF-κB p65 nuclear accumulation (Fig. 5A panels j–l). This was further supported by nuclear and cytoplasmic extracts in both HeLa (Fig. 5B) and MEF cells (Fig. 5C). We also examined the effect of ECTV and ECTV-Δ005 infection on the production of NF-κB-regulated transcripts. HeLa cells were mock-infected, or infected with ECTV or ECTV-Δ005 at a MOI of 5. At 12 hours post-infection, cells were stimulated with TNFα, and RNA samples were collected at 0, 2, 4, and 6 hours post-TNFα treatment. Samples were screened for the relative levels of RNA transcripts corresponding to TNFα, IL-1β, and IL-6; genes known to be upregulated by NF-κB [37]. All samples are presented as relative units compared to GAPDH as well as the unstimulated or 0 hour time point within each sample. Mock-infected cells displayed an increase in TNFα, IL-1β, and IL-6 transcript levels at 2 hours post-TNFα stimulation, as expected (Fig. 6). Transcript levels decreased at 4 and 6 hours post-stimulation, due to the up-regulation of NF-κB inhibitors such as IκBα (Fig. 1A) [38]. In contrast, infection with ECTV and ECTV-Δ005 prevented transcriptional upregulation of TNFα, IL-1β, and IL-6 (Fig. 6). We additionally screened our 0 hour time points to compare basal levels of NF-κB transcripts between samples (Fig. S5). This analysis, demonstrated that basal levels of TNFα, IL-1β and IL-6 were higher in infected cells compared to mock infected cells, however, we were still unable to detect any significant changes between cells infected with ECTV versus cells infected with ECTV-Δ005 (Fig. S5). These data correlated with our previous data indicating that infection with either ECTV or ECTV-Δ005 inhibited the nuclear accumulation of NF-κB p65.

**Figure 7:**
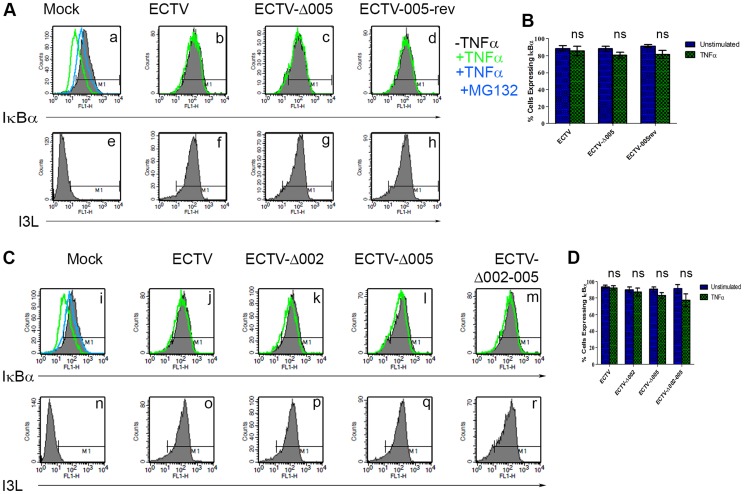
ECTV-Δ005, ECTV-Δ002 and ECTV-Δ002-005 inhibit TNFα induced IκBα degradation. (A) HeLa cells were mock-infected or infected with ECTV, ECTV-Δ005 or ECTV-005-rev at a MOI of 5. (C) Alternatively, HeLa cells were mock-infected or infected with ECTV, ECTV-Δ002, ECTV-Δ005 or ECTV-Δ002-005 at a MOI of 5. (A and C) At 12 hours post hours post-infection, cells were stimulated with 10 µM MG132 for 1 hour and hour and/or 10 ng ng/ml TNFα for 20 minutes. Cells were harvested, fixed and permeablized, followed by staining with anti minutes. Cells were harvested, fixed and permeablized, followed by staining with anti-IκBα and anti-I3L. Samples were subjected to flow cytometry, IκBα (A panels a–d, and C panels i–m) or I3L (A panels e–h and C panels n–r) are measured along the x-axis. (B and D) The percentage of cells expressing IκBα were measured and plotted as the average of three independent experiments with SEM.

Finally, we looked upstream at IκBα levels. HeLa cells were infected with ECTV, ECTV-Δ005, or ECTV-005-rev. At 12 hours post-infection, cells were stimulated with TNFα, fixed and stained with anti-IκBα or anti-I3L, an early poxvirus protein that is indicative of infection, and analyzed by flow cytometry. Unstimulated cells (shown in black) demonstrated high levels of IκBα that decreased following TNFα stimulation (shown in green) (Fig. 7A panel a). As expected, TNFα-stimulated mock-infected cells that were pre-treated with the proteasome inhibitor MG132 maintained IκBα levels (shown in blue) (Fig. 7A panel a). ECTV-infected cells stimulated with TNFα also indicated no change in the level of IκBα, lending further support that IκBα is not degraded in cells infected with ECTV (Fig. 7A panel b). Additionally, TNFα-stimulated cells infected with ECTV-Δ005 or ECTV-005-rev also inhibited IκBα degradation (Fig. 7A panels c and d). These data were quantified by measuring the percentage of cells with IκBα expression from three independent experiments to obtain standard errors (Fig. 7B). Overall, despite lacking EVM005, ECTV-Δ005 inhibits IκBα degradation.

Since EVM005 is one of four ankyrin/F-box proteins in ECTV, it is possible that deletion of more than one open reading frame may be necessary to render ECTV susceptible to TNFα-induced NF-κB activation and degradation of IκBα. Therefore, we used the Selectable and Excisable Marker system to excise four open reading frames, EVM002, EVM003, EVM004, and EVM005, from the left end of the ECTV genome (Fig. S3 and S4) [36]. Notably, EVM002 and EVM003 are duplicated genes that are encoded on both ends of the ECTV genome. ECTV-Δ002-005 is depleted of both copies of EVM002, but only the left end copy of EVM003 (Fig. S4). EVM002 is an ECTV-encoded ankyrin/F-box protein that interacts with the SCF ubiquitin ligase and inhibits NF-κB activation by interacting with NF-κB1/p105, a member of the IκB family of proteins [25], [31], [39]. Deletion of EVM002 from ECTV led to slightly increased NF-κB levels *in vivo*, contributing to decreased virulence, potentially through low level paracrine stimulation of interferon and NF-κB in neighbouring cells [40]. Significantly, deletion of the EVM002 ortholog, CPXV006, from CPXV, rendered CPXV susceptible to NF-κB activation [39]. EVM003 encodes a vTNFR, but a copy of this gene is present at both ends of the genome. Thus, even though EVM003 was deleted from the left end of the genome, EVM003 is still expressed from the right end (Fig. S4). EVM004 encodes a BTB-only protein with unknown function [41], [42]. We tested the ability of this virus, lacking two ankyrin/F-box proteins that inhibit NF-κB activation, to inhibit IκBα degradation. HeLa cells were mock-infected, or infected with ECTV, single deletion strains ECTV-Δ002, ECTV-Δ005, or the large deletion strain ECTV-Δ002-005, and analyzed for their ability to protect against TNFα-induced IκBα degradation using flow cytometry (Fig. 7C). Following stimulation with TNFα, ECTV, ECTV-Δ002, and ECTV-Δ005, inhibited IκBα degradation (Fig. 7C panels j–l). Additionally, IκBα was still protected from degradation in cells infected with ECTV-Δ002-005 (Fig. 7C panel m). As before, staining with anti-I3L indicated virus infection (Fig. 7C panels n–r). These data were quantified by measuring the percentage of cells with IκBα expression from three independent experiments to obtain standard errors (Fig. 7D). These data indicate that deletion of more than two ankyrin/F-box proteins, and potentially other ECTV encoded NF-κB inhibitors, may be necessary to render ECTV susceptible to TNFα-induced NF-κB activation.

### EVM005 is required for ECTV virulence in C57BL/6 and A/NCR mouse strains

**Figure 8:**
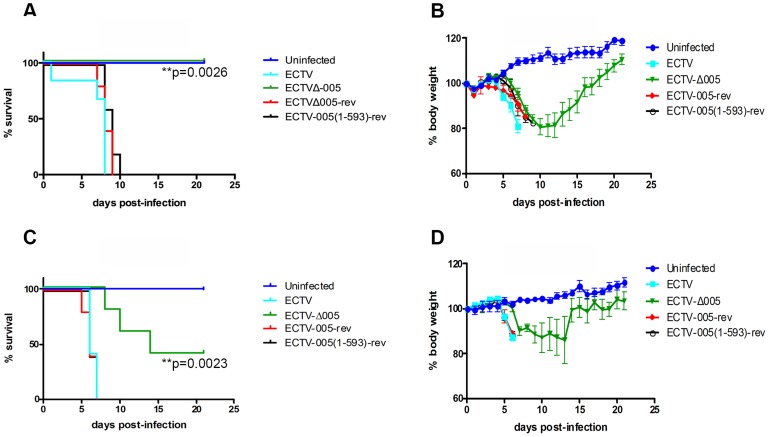
EVM005 is required for virulence during ECTV infection of C57BL/6 and A/NCR mice. (A and B) Female C57BL/6 mice were mock-infected or infected with 10,000 pfu of ECTV, ECTV pfu of ECTV, ECTV-Δ005, ECTV-005-rev or ECTV-005(1-593)-rev via intranasal inoculation. Mice were monitored daily for mortality, day of death (A), and body weight (B). (C and D) Alternatively, female A/NCR mice were mock-infected or infected with 10,000 pfu of ECTV, ECTV pfu of ECTV, ECTV-Δ005, ECTV-005-rev or ECTV-005(1-593)-rev via footpad injection. Mice were monitored daily for day of death and mortality (C) as well as body weight (D). Morality curves were statistically analyzed using a Log-rank (Mantel-Cox) test.

To determine if EVM005 was required for virulence, we used A/NCR or C57BL/6 mouse strains. A/NCR mice are highly susceptible to lethal infection by all evaluated routes, including the footpad, whereas C57BL/6 mice are only susceptible to lethal infection via the intranasal route [43]–[45]. Groups of five female C57BL/6 mice were mock-infected, or infected with 10-fold escalating doses of ECTV, ECTV-Δ005, ECTV-005-rev, or ECTV-005(1-593)-rev via the intranasal route with doses ranging between 10^2^ and 10^4^ pfu (Fig. S6). Following infection, body weight and mortality were monitored daily. Data from one challenge dose is displayed (Fig. 8A and B). C57BL/6 mice infected with ECTV, ECTV-005-rev, or ECTV-005(1-593)-rev succumbed to infection between day seven and ten; however, mice infected with ECTV-Δ005 survived through day 21 (Fig. 8A). C57BL/6 mice infected with ECTV-Δ005 displayed an initial weight loss through day 13, followed by weight gain similar to naive mice by day 21 (Fig. 8B).

We also assessed the contribution of EVM005 to virulence in the A/NCR mouse strain [44], [45]. Five female A/NCR mice were mock-infected, or infected with ECTV, ECTV-Δ005, ECTV-005-rev, or ECTV-005(1-593)-rev via the footpad [44]. We infected sets of five mice with escalating 10-fold doses between 10^1^ and 10^4^ pfu per mouse and monitored daily changes in body weight, day of death and mortality (Fig. S7). Data from one challenge dose is displayed (Fig. 8C and D). Similar to the data observed in C57BL/6 mice (Fig. 8A and B), ECTV-Δ005 was attenuated in A/NCR mice compared to wild type ECTV, ECTV-005-rev, and ECTV-005(1-593)-rev (Fig. 8C and D). The data demonstrated that mice infected with ECTV, ECTV-005-rev, and ECTV-005(1-593)-rev succumbed to infection by day 7 post-infection. Alternatively, two of five mice infected with ECTV-Δ005 survived through day 21 (Fig. 8C and D). Together, the results suggest that EVM005 is a critical virulence factor for infection of two mouse strains by two different routes of infection. Notably, mice infected with ECTV-005(1-593)-rev displayed similar mortality and weight loss profiles to mice infected with wild type ECTV and ECTV-005-rev, suggesting that although the F-box domain was necessary for inhibition of the NF-κB pathway *in vitro*, the ankyrin domains alone are sufficient for virulence.

### Mice infected with ECTV-Δ005 demonstrate decreased viral spread and increased immune cell activation

**Figure 9:**
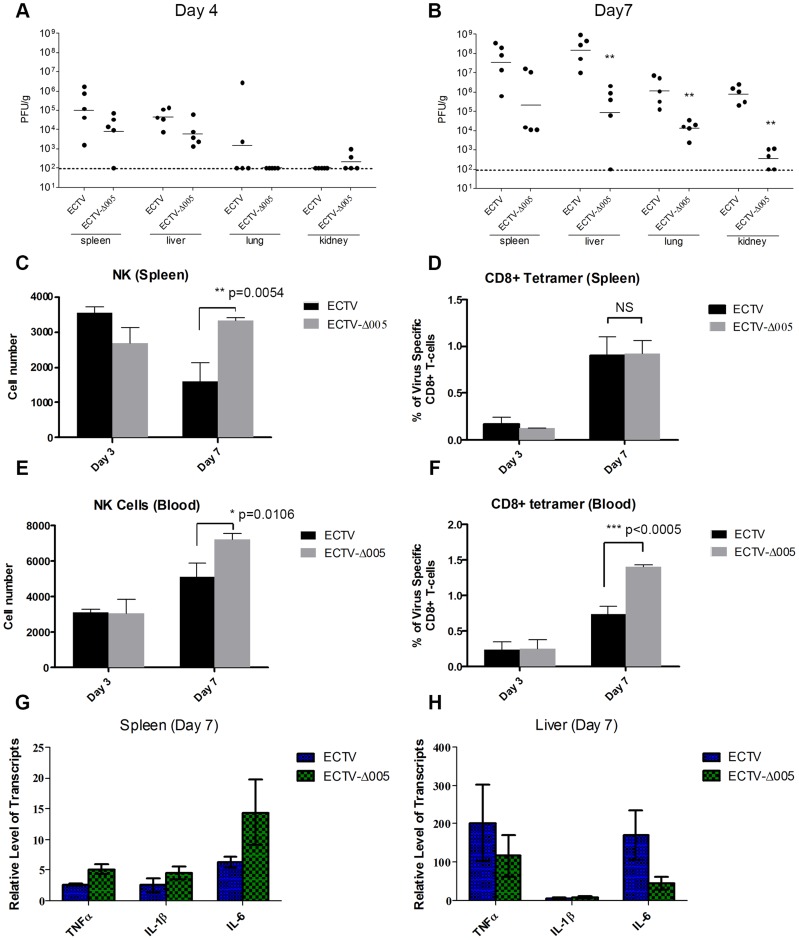
EVM005 inhibits activation of the immune response. C57BL/6 mice were infected with 10,000 pfu of ECTV or ECTV pfu of ECTV or ECTV-Δ005 via intranasal inoculation. Tissues and whole blood were collected at 2, 3, 4 and 7 days post infection. (A and B) Spleen, liver, lung and kidney tissues were homogenized and plated onto BSC-1 cells to measure viral titers at day 4 (A) and 7 (B) post infection. Cell suspensions were prepared from infected spleens (C and D) or peripheral blood (E and F) on day 3 and 7 and analyzed by flow cytometry. (C and E) NK cells are identified as CD45+, CD3−, and NK1.1+. We gated 50,000 CD45+ cells, and presented the total number of NK1.1+ cells within this lymphocyte population (D and F) Virus-specific CD8+ T-cells were identified as CD8+, CD45+, and tetramer+, and presented as the ratio of tetramer+ cells to total CD8+ T-cells within each sample. (G and H) RNA was purified from spleen (G) and liver (H) and subjected to qRT-PCR to quantify transcriptional upregulation of TNFα, IL-1β and IL-6 at 7 days post infection. Data are normalized against GAPDH.

Though EVM005 was a potent inhibitor of NF-κB activation in tissue culture, deletion of EVM005 did not abrogate the ability of ECTV to prevent activation of NF-κB. Since tissue culture assays lack many components of the immune response, we wanted to explore the contribution of EVM005 to immune inhibition and virulence *in vivo*. To monitor virus spread and activation of the immune response, C57BL/6 mice were infected via intranasal inoculation with ECTV or ECTV-Δ005, and sacrificed at days 3, 4, and 7 post-infection (Fig. 9). Tissue from the spleen, liver, lungs, and kidneys was harvested, and the amount of virus present was determined by plaque assay (Fig. 9A and B). At 4 days post-infection, ECTV and ECTV-Δ005 showed no significant difference in growth rate in all organs tested (Fig. 9A); however, at 7 days post-infection, ECTV had grown to significantly higher levels than ECTV-Δ005 in lung, kidney and liver tissues (Fig. 9B). Notably, the decrease in viral spread correlates well with the decreased mortality previously described (Fig. 8A).

To measure the immune response, whole blood and splenocytes were harvested on days 3 and 7 post-infection and immune cell populations were quantified using flow cytometry (Fig. 9C–F). In mice infected with ECTV-Δ005, there was a significant increase in circulating and splenic NK cells at day 7 compared to ECTV-infected mice (Fig. 9C and E). Additionally, we observed a significant increase in circulating virus-specific CD8+ T-cells at day 7 post-infection in mice infected with ECTV-Δ005 compared to those infected with ECTV (Fig. 9F). Notably, we did not observe an increase in virus-specific CD8+ T-cells in the spleen on day 7 (Fig. 9D). The data suggest that the virus-specific CD8+ T-cells are being activated and expanded in non-splenic tissues before entering circulation. Finally, we assayed for transcriptional upregulation of NF-κB-regulated genes in liver and spleen on day 7 post-infection (Fig. 9G and H). Transcriptional upregulation of TNFα, IL-1β, and IL-6 was determined by harvesting RNA from tissue samples and subjecting it to qRT-PCR. Mice infected with ECTV-Δ005 did not demonstrate an increase in NF-κB-regulated transcripts compared to ECTV-infected mice. These observations support a role for EVM005 in regulating virulence that is independent of its ability to inhibit NF-κB activation. Together these data indicate that mice infected with ECTV-Δ005 displayed boosted immune cell repertoires, increased viral clearance, and decreased mortality compared to mice infected with wild type ECTV.

## Discussion

The NF-κB family of transcription factors regulate a variety of genes involved in inflammation and innate immunity [1]. Not surprisingly, viruses have evolved multiple mechanisms to regulate NF-κB [3], [46], and a growing number of poxviral NF-κB inhibitors can be added to this list [5]. Previously, we identified four ankyrin/F-box proteins in ECTV that interact with the SCF ubiquitin ligase via C-terminal F-box domains; potentially recruiting a unique set of proteins to the SCF ubiquitin ligase [25]. The NF-κB signalling pathway is dependent on the SCF ubiquitin ligase for ubiquitination and degradation of the inhibitory protein, IκBα [47]. Here we demonstrate that IκBα is phosphorylated but not degraded during ECTV infection, suggesting that signalling is inhibited at the point of IκBα ubiquitination, an event mediated by the SCF ubiquitin ligase (Fig. 1). Additionally, we demonstrate that the ECTV-encoded ankyrin/F-box protein, EVM005, inhibits p65 nuclear accumulation and IκBα degradation in a process that requires its C-terminal F-box domain (Fig. 3 and 4). From this, we conclude that EVM005 is an inhibitor of NF-κB activation through manipulation of the SCF ubiquitin ligase. ECTV lacking the EVM005 open reading frame, ECTV-Δ005, was created and tested for its ability to inhibit NF-κB activation (Fig. 5–7). Even though EVM005 was deleted from the genome, ECTV-Δ005 still inhibited p65 nuclear accumulation (Fig. 5), the production of NF-κB-regulated transcripts (Fig. 6), and degradation of IκBα in tissue culture (Fig. 7). Significantly, ECTV lacking EVM005 was attenuated in both A/NCR and C57BL/6 mouse strains, indicating an additional NF-κB-independent mechanism for EVM0005 (Fig. 8). Interestingly, ECTV expressing a mutant of EVM005 lacking the F-box domain was still virulent, demonstrating that the ankyrin domains alone were sufficient for virulence (Fig. 8). Mice infected with ECTV devoid of EVM005 were able to mount a stronger immune response, consisting of higher numbers of NK cells and virus-specific CD8+ T-cells (Fig. 9). A strong immune response is most likely responsible for virus clearance and decreased mortality of mice, and the observed decrease in virus spread to the liver, lungs, spleen, and kidneys (Fig. 9).

EVM005 is one of many open reading frames encoded by ECTV that inhibits NF-κB activation. Given the plethora of poxvirus-encoded inhibitors of NF-κB, the deletion of multiple open reading frames is likely required to render ECTV susceptible to NF-κB activation. In an attempt to create a strain of ECTV that was unable to inhibit TNFα-induced NF-κB activation, we deleted four open reading frames from the left end of the ECTV genome, including EVM002, EVM003, EVM004, and EVM005 [36]. Large deletion strains of VACV, such as VACV811, and modified vaccinia virus Ankara (MVA), have been tremendous tools for the characterization of novel poxvirus-host interactions [48], [49]. Although VACV811 is missing 55 open reading frames, this virus is capable of inhibiting NF-κB activation [50]. MVA is an attenuated strain of VACV that has been passaged over 500 times in chicken embryonic fibroblasts and has acquired numerous gene deletions, truncations, and point mutations [49]. MVA is the only large deletion virus that has been rendered susceptible to TNFα induced NF-κB activation [51]. Our large deletion strain of ECTV, ECTV-Δ002-005, was able to inhibit TNFα-induced IκBα degradation (Fig. 7C and D). EVM002 is an ankyrin/F-box protein that we have previously shown to interact with the SCF ubiquitin ligase and inhibit p65 nuclear accumulation [25], [31]. These data suggest that deletion of more than two ankyrin/F-box proteins, and potentially other ECTV-encoded inhibitors of NF-κB activation, would be required to render ECTV susceptible to NF-κB activation. Creation of an ECTV strain unable to inhibit NF-κB activation would allow us to investigate how ECTV infection triggers NF-κB activation, since little is known about how poxviruses activate this pathway.

Regulation of the NF-κB pathway by poxviruses has been investigated, and a variety of unique NF-κB inhibitors have been found [5], [46]. These inhibitors include poxvirus-secreted proteins, such as the soluble vTNFR and vIL-1R [13]–[15], as well as eight VACV-encoded proteins that act within the cell, including M2, K1, B14, N1, K7, A46, A49, and A52 [16]–[22], [24]. Of the known virus encoded inhibitors of NF-κB, only K1, N1 and A46 contain orthologs in ECTV [16], [17], [19]–[22]. That ECTV is missing many NF-κB inhibitors is perhaps what contributes to the variation observed in phospho-IκBα accumulation between ECTV and VACV, where VACV-infected HeLa cells showed lower levels of phospho-IκBα accumulation, and accumulation was delayed in comparison to ECTV-infected cells (Fig. 1A). This observation may be linked to the additional upstream inhibitors encoded by VACV.

Our data demonstrate an accumulation of phospho-IκBα in ECTV-infected cells that is linked to regulation of the cellular SCF ubiquitin ligase by poxviral ankyrin/F-box proteins. The cellular F-box protein, β-TRCP, recognizes phospho-IκBα in uninfected cells and mediates ubiquitination and subsequent degradation via the 26S proteasome [52]. Though ECTV encodes four ankyrin/F-box proteins [25], [34], we tested the ability of EVM005 to inhibit NF-κB signalling, since it is unique to ECTV and CPXV [25]. Our data demonstrate that EVM005 inhibited IκBα degradation, perhaps through competition with β-TRCP for available Skp1 binding sites at the SCF ubiquitin ligase. This competition would disrupt the association between Skp1 and β-TRCP, an interaction that is required for IκBα ubiquitination and degradation. This idea is consistent with our data demonstrating the requirement of the F-box domain by EVM005 in order to inhibit degradation of IκBα (Fig. 4 and 5). In a similar fashion, HIV-encoded Vpu disrupts the association between β-TRCP and Skp1, thus inhibiting the ubiquitination and degradation of IκBα [53]. Notably, the VACV protein A49 inhibits NF-κB signalling by binding to β-TRCP in a similar fashion to Vpu [24]. This represents a fascinating example of convergent evolution, since both EVM005 and A49 serve similar functions to inhibit NF-κB signalling, but by targeting different proteins within the SCF ubiquitin ligase. Notably, an EVM005 ortholog is not encoded by VACV, and A49 is not encoded by ECTV, demonstrating the importance of regulating NF-κB through the SCF ubiquitin ligase. Of note, our data do not rule out the possibility that EVM005 recruits substrates that are involved in NF-κB activation for ubiquitination; however, we were unable to detect degradation of IκBα, NF-κB1 p50/105, or p65 in ECTV-infected cells (N. van Buuren and M. Barry unpublished data).

Regulation of NF-κB activation by poxviral ankyrin/F-box proteins has been investigated for ECTV-encoded EVM002, CPXV-encoded proteins, CP77 and CPXV006, and the variola protein, G1R [29], [31], [39], [40]. Similar to EVM005, these proteins interact with the cellular SCF ubiquitin ligase [29], [31]. In contrast to EVM005, the mechanism by which G1R inhibits NF-κB activation does not depend on the F-box domain [31], [39]. Instead, G1R and its orthologs, CPXV006 and EVM002, bind to the N-terminus of p105, an inhibitory protein similar to IκBα, to prevent TNFα-induced degradation [31]. Degradation of p105 is generally mediated by the SCF^β-TRCP^ ubiquitin ligase following TNFα stimulation, similar to IκBα [54]. Deletion of CPXV006, a G1R ortholog encoded by CPXV, rendered CPXV susceptible to NF-κB activation [39]. Additionally, ECTV lacking EVM002 demonstrated decreased virulence and slightly increased levels of NF-κB activation *in vivo* [40]. We demonstrated that ECTV lacking EVM002 still inhibited IκBα degradation in tissue culture, demonstrating that the ECTV ankyrin/F-box proteins act collectively to inhibit IκBα degradation (Fig. 7C and D). In contrast, ECTV lacking EVM005 was still a potent inhibitor of NF-κB activation in culture and *in vivo* (Fig. 5–7, and 9). CP77 contains a shortened F-box domain that is necessary to inhibit NF-κB activation [29]. Additionally, CP77 binds to free p65 through its ankyrin repeat domains. The model suggests that CP77 replaces the regulatory protein IκBα, following its degradation, holding the NF-κB transcription factor, p65, inactive in the cytoplasm [29]. In contrast, we were unable to detect an interaction between EVM005 with p65, p50/105 or IκBα (N. van Buuren and M. Barry, unpublished data). Similar to our data with EVM005, CP77 serves a dual role for CPXV as a host range factor in addition to its role in the inhibition of NF-κB activation [55], [56]. It is clear that the ankyrin domains play a major role for most of the ankyrin/F-box proteins described to date, and this is consistent with the virulent phenotype of ECTV-005(1-593)-rev (Fig. 8). Discovery of binding partners for the ankyrin domains of EVM005 will likely provide insight to the mechanism of virulence controlled by EVM005. Together, the data suggest that the poxvirus encoded ankyrin/F-box proteins possess unique mechanisms to regulate NF-κB activation.

Although poxviral ankyrin/F-box proteins associate with Skp1 in the SCF ubiquitin ligase through their F-box domains, identification of substrates recruited for ubiquitination has eluded the field. Additionally, of the sixty-nine cellular F-box proteins encoded in the human genome, substrates have been identified for only nine [57]. The poxviral F-box proteins are suspected to function as substrate adaptor molecules for the SCF ubiquitin ligase, using their unique ankyrin domains to recruit still unidentified cellular or viral target proteins. Although binding partners, other than Skp1, have been identified for several of the poxvirus ankyrin/F-box proteins, none of these identified proteins have been characterized as *bona fide* substrates for ubiquitination [30], [31], [55]. In support of the substrate hypothesis, the ankyrin-only mutant virus, ECTV-005(1-593)-rev, was still virulent, supporting a critical role for the ankyrin domains, potentially in substrate recruitment. The data presented in this paper suggest a mechanism in which EVM005 inhibits degradation of cellular substrates, such as IκBα. This suggests an alternative mechanism for the poxviral ankyrin/F-box proteins as inhibitors of the SCF ubiquitin ligase.

Finally, we determined that EVM005 was a required virulence factor for ECTV during infection of C57BL/6 and A/NCR mice. However, ECTV-Δ005 was capable of inhibiting IκBα degradation, p65 nuclear accumulation and the synthesis of NF-κB regulated transcripts (Fig. 5, 6 and 7). These data suggest that an EVM005 function independent of NF-κB inhibition is responsible for mediating virulence during ECTV infection. To this end, we demonstrated that ECTV-Δ005 spread *in vivo* was suppressed compared to ECTV and that this observation correlated with increased immune cell activation (Fig. 9). It is possible that EVM005 regulates the immune response *in vivo*. At this time, any regulation of the immune response appears to be independent of NF-κB activation as we were unable to detect increased transcription of TNFα, IL-1β or IL-6 in spleens or livers of infected mice on day 7 (Fig. 9). We hypothesize that EVM005 is recruiting substrates to the SCF ubiquitin ligase through its ankyrin domains. Infection of mice with ECTV-005(1-593)-rev demonstrated that expression of the ankyrin domains alone was sufficient for virulence in both the A/NCR and C57BL/6 mice. Potentially the ankyrin-only mutant is still able to bind and sequester these hypothetical SCF substrates and that sequestration alone was sufficient for virulence. If the poxvirus-encoded ankyrin/F-box proteins truly function as substrate adaptors for the cellular SCF ubiquitin ligase, the identification of substrates through proteomics approaches could lead to insight into how EVM005 aids in virulence. Additionally, as mice infected with ECTV-Δ005 demonstrated increased immune responses, we feel that it is therefore likely that these hypothetical target substrates function in immune cell regulation.

In conclusion, our data show that ECTV-encoded EVM005 is a unique inhibitor of NF-κB activation and also suggests the existence of an NF-κB-independent mechanism for EVM005 to contribute to virulence and inhibition of immune activation. In contrast to previously characterized poxviral inhibitors of NF-κB, EVM005 requires its C-terminal F-box domain to manipulate the cellular SCF ubiquitin ligase and inhibit IκBα degradation. Further characterization of the NF-κB-independent mechanism of virulence mediated by EVM005 as well as the identification of ubiquitinated substrate proteins remains a goal of our laboratory.

## Materials and Methods

### Cells and viruses

HeLa, mouse embryonic fibroblast (MEF), and Baby Green Monkey Kidney (BGMK) cells were obtained from the American Type Culture Collection. U20S-Cre cells were generously provided by Dr. John Bell (University of Ottawa, Ottawa, Canada). HeLa and U20S-Cre cells were cultured in Dulbecco's Modified Eagle Medium (DMEM) supplemented with 10% fetal bovine serum, 50 U/ml of penicillin, 50 µg/ml of streptomycin and 200 µM glutamine (Invitrogen Corporation). MEF cells were cultured in Dulbecco's Modified Eagle Medium (DMEM) supplemented with 10% fetal bovine serum, 50 U/ml of penicillin, 50 µg/ml of streptomycin, 200 µM glutamine, and 10 µM non-essential amino acids (Invitrogen Corporation). BGMK cells were cultured in DMEM supplemented with 10% newborn calf serum, 50 U/ml of penicillin, 50 µg/ml of streptomycin and 200 µM glutamine. Vaccinia virus strain Copenhagen (VACV), and ectromelia virus strain Moscow (ECTV) were propagated in BGMK cells and harvested as previously described [58].

### Plasmid constructs

Construction of pcDNA3-Flag-EVM005 and pcDNA3-Flag-EVM005(1-593) were previously described [25]. Construction of pDGloxP-EVM005KO was performed by amplification of 150 base pairs of DNA upstream and downstream of EVM005 from ECTV genomic DNA using Taq polymerase (Invitrogen Corporation). The upstream region of homology was amplified with the following forward 5′-(*Hin*dIII)- AAGCTTCTCTACAAAGTATAATATATT-3′ and reverse 5′-(*Xho*I)- CTCGAGATATTATACATATTAGATGTG-3′ primers. The downstream region of homology was amplified using the following forward 5′-(*Not*I)- GCGGCCGCTCGT ACCCGCGAACAAAATAG-3′ and reverse 5′-(*Bam*HI)- GGATCCTTTTTTATAAACGATA TTGTT-3′ primers. The 150 base pair fragments were cloned into pGEM-T (Promega). The upstream region of homology was subcloned in to the pDGloxP vector using *Xho*I and *Hin*dIII restriction sites. The downstream region of homology was subcloned into the *Bam*HI and *Not*I restriction sites, to create pDGloxP-EVM005KO. To clone pGEMT-EVM005-reverant the forward, 5′-ATCAATGGCCGTCTCGAT-3′, and reverse 5′-AAGAAACAAGATACAAGA-3′ primers were used to amplify a 2787 bp PCR product from wild type ECTV viral genomic DNA using LongAmp *Taq* (New England Biolabs). The resulting PCR product was subsequently cloned into pGEMT (Promega). To clone pDG-loxP-EVM002KO, 150 bp of DNA at the 5′ end of the EVM002 open reading frame were amplified by PCR using *Taq* polymerase (Invitrogen Corporation) and the forward primer, 5′-(*Hin*dIII)- AAGCTTCTCATAATGATTTACTTTTTC-3′ and the reverse primer, 5′-(*Xho*I)- CTCGAGCGATTCCGTCCAAGATGATAA-3′. The 150 bp of DNA at the 3′ end of the EVM002 open reading frame were amplified with the forward primer, 5′-(*Not*I)- GCGGCCGCGGTGCTATATCTTTTCCGTTT-3′, and the reverse primer, 5′-(*Bam*HI)- GGATCCTAGAAAGAAAATATTTAAAAA-3′. The 5′ and 3′ 150 bp regions of homology were TA cloned into pGEMT (Promega) following PCR. The 5′ and 3′ regions of homology were then subcloned one at a time into the pDGloxPKO vector using *Hin*dIII and *Xho*I, followed by *Bam*HI and *Not*I, for the 5′ and 3′ sides, respectively.

### Construction of ECTV recombinant viruses

BGMK cells were infected with ECTV at a MOI of 0.01 and transfected with 10 µg of linearized pDGloxP-EVM005KO or pDGloxP-EVM002KO using Lipofectamine 2000 (Invitrogen Corporation) [35], [36]. Recombinant ECTV-Δ005-YFP-GPT or ECTV-Δ002-YFP-GPT were selected in BGMK media containing 250 µg/ml xanthine (Sigma-Aldrich), 15 µg/ml hypoxanthine (Sigma-Aldrich), and 25 µg/ml mycophenolic acid (MPA) (Sigma-Aldrich). Drug resistance and YFP fluorescence were used to select recombinant viruses. Removal of the *yfp-gpt* marker cassette from ECTV-Δ005-YFP-GPT or ECTV-Δ002-YFP-GPT was performed using U20S cells stably expressing a cytoplasmic mutant of the Cre recombinase (U20S-Cre) (provided by Dr. J. Bell, University of Ottawa). White ECTV foci, lacking YFP-GPT expression were selected and purified to create ECTV-Δ005 and ECTV-Δ002. ECTV-005-rev and ECTV-005(1-593)-rev were cloned by infecting BGMK cells with ECTV-Δ005-YFP-GPT at an MOI of 0.01 followed by transfection with pGEMT-EVM005-rev or pGEMT-EVM005(1-593)-rev plasmids. Infected cells were harvested at 48 hours post-infection and recombinant ECTV-005-rev or ECTV-005(1-593)-rev were selected through lack of YFP fluorescence using a fluorescent inverted microscope and a FITC filter (Leica).

PCR analysis of viral genomes verified insertion and deletion of the *yfp-gpt* cassette. Taq polymerase and forward 5′-(HindIII)- AAGCTTCTCTACAAAGTATAATATATT-3′ and reverse 5′-(BamHI)- GGATCCTTTTTTATAAACGATATTGTT-3′ primers were used to amplify the EVM005 locus. A multi-step growth curve was used to analyze the growth of ECTV-Δ005, ECTV-005-rev and ECTV-005(1-593)-rev on BGMK cells. BGMK cells were infected with ECTV, ECTV-Δ005, ECTV-005-rev or ECTV-005(1-593)-rev at an MOI of 0.05 to perform the multi-step growth curve. Virus growth was assayed using plaque assays from samples collected at indicated time points.

To create the large deletion strain of ECTV, lacking EVM002, EVM003, EVM004 and EVM005 from the left end of the genome, we used sequential insertion and Cre-mediated excision of the *yfp-gpt* cassette (Fig. S3). Following Cre-mediated excision, one residual loxP site remains in place of the *yfp-gpt* cassette. BGMK cells were infected with ECTV-Δ002 at a MOI of 0.01 and transfected with 10 µg of linearized pDGloxP-EVM005KO using Lipofectamine 2000 (Invitrogen Corporation). YFP-GPT positive virus was selected as described above to create ECTV-Δ002/Δ005-YFP-GPT. To delete EVM002, EVM003, EVM004 and EVM005, U20S-Cre cells were infected with ECTV-Δ002/Δ005-YFP-GPT at a MOI of 0.01 and white foci were selected using immunofluorescence. The resulting virus, ECTV-Δ002-005, lacks all sequences between the residual loxP site in the EVM002 locus and the new loxP site at the right side of the EVM005 locus introduced during recombination with pDGloxP-EVM005KO. PCR analysis of the EVM002, EVM003, EVM004 and EVM005 loci confirmed the identity and purity of this large deletion strain of ECTV (Fig. S4).

### Antibodies

Mouse and rabbit anti-Flag M2 were purchased from Sigma-Aldrich, anti-poly(ADP-ribose) polymerase (PARP) was purchased by (BD Biosciences) and anti-β-tubulin was purchased from ECM Biosciences. Antibodies specific for Skp1 and I5L were previously described [25], [59] Antibodies recognizing the early poxvirus protein I3L were generously donated by Dr. David Evans (University of Alberta). Antibodies recognizing IκBα and phospho-IκBα were purchased from Cell Signalling Technologies. Anti-NF-κB p65 was purchased from Santa Cruz Biotechnology. Antibodies that detected cell surface markers CD45, NK1.1, CD3, and CD8 were purchased from BD Biosciences. An APC labeled tetramer specific to the immunodominant epitope for VACV B8R/CD8 T cell expression was obtained from the NIH tetramer facility.

### Cytoplasmic and nuclear extracts

HeLa cells or MEF cells were mock-infected or infected with ECTV, VACV, or ECTV-Δ005 at a MOI of 5 for 12 hours followed by stimulation with either 10 ng/ml TNFα (Roche) or 10 ng/ml IL-1β (PeproTech Inc) for 20 minutes. Cells were harvested and lysed in cytoplasmic extract buffer containing 10 mM HEPES, 10 mM KCl, 0.1 mM EDTA (pH 8.0), 0.1 mM EGTA (pH 8.0), 1 mM dithiolthreitol (DTT) and 0.05% NP40. Samples were centrifuged at 1000× *g* for five minutes to remove nuclei. Supernatants were collected and resuspended in SDS sample buffer. The nuclear pellets were washed and resuspended in nuclear extract buffer containing 20 mM HEPES, 25% glycerol, 0.4M NaCl, 1 mM EDTA (pH 8.0), 1 mM EGTA (pH 8.0), and 1 mM DTT and lysis was performed on ice for 30 minutes. Samples were centrifuged at 1000× *g* for five minutes. Supernatants were collected as nuclear extracts and mixed with SDS sample buffer.

### Immunofluorescence microscopy

HeLa cells were mock-transfected or transfected with pcDNA3-Flag-EVM005 or pcDNA3-Flag-EVM005(1-593). Alternatively, HeLa cells were mock-infected or infected with ECTV, VACV, or ECTV-Δ005 at a MOI of 5. At 12 hours post-infection or transfection, cells were stimulated with 10 ng/ml TNFα (Roche) or 10 ng/ml IL-1β (PeproTech Inc) for 20 minutes and fixed with 2% paraformaldehyde (Sigma-Aldrich) for 10 minutes at room temperature. Cells were permeablized with 1% NP40 and blocked with 30% goat serum (Invitrogen Corporation). Cells were stained with anti-NF-κB p65 (1∶200) alone or co-stained with either anti-NF-κB p65 (1∶200) and mouse anti-Flag M2 (1∶200), or anti-IκBα (1∶125) and rabbit anti-Flag M2 (1∶200). Cells were stained with secondary antibodies anti-mouse-AlexaFluor488 and anti-rabbit-AlexaFluor546 at a dilution of 1∶400 (Jackson Laboratories). Coverslips were mounted using 4 mg/ml N-propyl-gallate (Sigma Aldrich) in 50% glycerol containing 250 µg/ml 4′,6-diamino-2-phenylindole (DAPI) (Invitrogen Corporation) to visualize nuclei. Cells were visualized using the 40× oil immersion objective of a Ziess Axiovert 200M fluorescent microscope outfitted with an ApoTome 10 optical sectioning device (Ziess). To quantify the number of cells displaying a nuclear localization of p65 greater than 50 cells were counted in three independent experiments.

### Flow cytometry

HeLa cells were transfected with pcDNA3-Flag-EVM005 or pcDNA3-Flag-EVM005(1-593) using Lipofectamine 2000 (Invitrogen Corporation). Alternatively, HeLa cells were mock-infected or infected with ECTV, ECTV-Δ002, ECTV-Δ005, ECTV-005-rev, or ECTV-Δ002-005 at a MOI of 5. At 24 hours post-transfection or 12 hours post infection, mock-infected cells were stimulated with 10 µM MG132. Samples were then left unstimulated or stimulated with 10 ng/ml TNFα (Roche) or 10 ng/ml IL-1β (PeproTech Inc) for 20 minutes. Cells were fixed in 0.5% paraformaldehyde (Sigma-Aldrich) for 15 minutes at 37°C. Cells were permeablized with ice cold 90% methanol for 30 minutes. Transfected cells were co-stained with rabbit anti-Flag M2 (1∶200) and anti-IκBα (1∶400). Cells were stained with anti-rabbit phycoerythrin (1∶1000) and anti-mouse-AlexaFluor488 (1∶400) (Jackson Laboratories) secondary antibodies, and resuspended in PBS. Infected cells were stained with anti-I3L (1∶100) or anti-IκBα (1∶400), followed by anti-mouse-AlexaFluor 488 (1∶400). Data were collected on a Becton Dickinson FACScan flow cytometer and analyzed with CellQuest software. Mean fluorescence intensities were calculated for three independent experiments.

Whole blood or splenocytes were harvested on days 3 or 7 post infection from C57BL/6 mice infected with ECTV or ECTV-Δ005. Whole blood was lysed with water at a 40∶1 water to blood volume ratio for ten seconds then brought to 1X with 10X PBS. The remaining white blood cells were resuspended in PBS with 2% FBS prior to staining. Spleen tissues were disrupted with the Bullet Blender (STL Scientific) for ∼2 minutes at room temperature using the lowest setting in PBS. The cell suspension was pelleted and the red blood cells were lysed with BD Pharm Lyse. The remaining white blood cells were resuspended in PBS with 2% FBS prior to staining. Cells were stained for flow cytometry using Fc block and the described antibody cocktails in PBS with 2% FBS for 20–30 minutes on ice. Cells were washed twice with PBS containing 2% FBS then fixed on ice with PBS containing 2% FBS and 1% methanol free formaldehyde. Stained cells were analyzed on a BD LSRII or BD Canto. NK cells were identified as being CD45 positive, CD3 negative and NK1.1 positive. These are defined in the literature as NK lytic cells and can only be identified in C57BL/6 mice [60]. An APC labeled tetramer specific to the immunodominant epitope for VACV B8R/CD8 T cell expression was obtained from the NIH tetramer facility [61]. Virus-specific CD8+ T-cells were identified as CD45 positive, CD8 positive, and tetramer positive.

### Real time PCR

HeLa cells were mock-infected or infected with ECTV or ECTV-Δ005. At 12 hours post infection cells were stimulated with 10 ng/ml TNFα and RNA was harvested using Trizol according to the manufacturer's protocol (Invitrogen Corp.). RNA samples were converted to cDNA using Superscript II reverse transcriptase (Invitrogen Corp.). Transcript levels were analyzed by real time PCR using the following primers, TNFα forward, 5′-GGCGTGGAGCTGAGAGATAAC-3′ and reverse, 5′-GGTGTGGGTGAGGAGCACAT-3′, IL-1β forward, 5′-TTCCCAGCCCTTTTGTTGA-3′ and reverse 5′-TTAGAACCAAATGTGGCCGTG-3′, IL-6 forward 5′-GGCACTGGCAGAAAACAACC-3′ and reverse 5′-GCAAGTCTCCTCATCGAATCC-3′ and GAPDH forward 5′-AGCCTTCTCCATGGTGGTGAAGAC-3′ and reverse 5′-CGGAGTCA ACGGATTTGGTCG-3′. Real time PCR was performed using the Sybr-Green master mix (Promega) and a MyIQ (BioRad) thermocycler. Data analysis was performed with IQ-5 software (BioRad). Data was presented as the average of three independent experiments.

Additionally, we measured transcriptional activation of TNFα, IL-1β and IL-6 in infected liver and spleen through isolation of RNA using Trizol (Invitrogen Corporation) as per manufacturer's protocol. RNA samples were subjected to qRT-PCR as described above to quantify transcriptional upregulation with reference to GAPDH.

### Reverse transcription PCR to detect viral gene expression

RNA Transcripts for EVM004, EVM005, EVM058, and GAPDH are analysed as described previously [41]. cDNA was used as a template and gene-specific primers were used to amplify the last 250 nucleotides (at the 3′ end) of each open reading frame. Transcripts were generated with the following primers: ECTV004 forward 5′-GTTTAATATCATGAACTGCGACTATCT-3′, and reverse, 5′-TTAATAATACCTAGAAAATATTCCACGAGC-3′, ECTV005 forward, 5′-TAGTGGTATTAGAGAGAAATGCAATCT-3′, and reverse, 5′-TCATTCATGTGTCTGTGTTTG-3′, I5L forward, 5′ATGGCGGATGCTATAACCGTT-3′, and reverse, 5′-TTAACTTTTCATTAATAGGGA-3′.

### Infection of C57BL/6 and A/NCR mice with ECTV

To determine the role of the ECTV encoded EVM005 in virulence we infected female C57BL/6 mice. Four to six week old female C57BL/6 mice were obtained from the National Cancer Institute (Frederick, MD). Groups of five mice were infected via the intranasal route with 10-fold escalating doses of wild type ECTV, ECTV-Δ005, ECTV-005-rev or ECTV-005(1-593)-rev. Mice were anesthetized with 0.1 ml/10 g body weight with ketamine HCL (9 mg/ml) and xylazine (1 mg/ml) by intraperitoneal injection. Anesthetized mice were laid on their dorsal side with their bodies angled so that the anterior end was raised 45° from the surface; a plastic mouse holder was used to ensure conformity. Strains of ECTV were diluted in PBS without Ca^2+^ and Mg^2+^ to the required concentration and slowly loaded into each naris (5 µl/naris). Mice were subsequently left *in situ* for 2 to 3 minutes before being returned to their cages. Mice were monitored for body weight daily for up to 21 days. Mice that demonstrated a drop in body weight to 70% of their original mass, or signs of severe morbidity were euthanized. To determine organ titers and immune activation, mice were sacrificed at 2, 3, 4, and 7 days post-infection, and tissue from the spleen, liver, lungs, and kidney were harvested in addition to blood collected by a needle stick in the heart. Tissue was homogenized using a tissue homogenizer (Next Advance), followed by dilution in PBS (10% w/v). Viral titers were determined on BSC-1 cells using a plaque assay. To prevent avoidable suffering, mice demonstrating a drop in body weight to 70% of their original mass, or signs of severe morbidity, were euthanized.

Alternatively, we infected the susceptible A/NCR strain of mice to determine the role of EVM005 during an ECTV infection. Five to ten week old female A/NCR mice were obtained from the National Cancer Institute (Frederick, MD). Groups of five mice with similar body mass were arranged into separate cages. Mice were anesthetized with CO_2_/O_2_ prior to infection. ECTV, ECTV-Δ005, ECTV-005-rev, and ECTV-005(1-593)-rev were diluted in PBS without Ca^2+^ and Mg^2+^ to the required concentration and 10 µl was used to infect mice via footpad injection. Body weight, day of death and mortality were monitored daily. Mice that demonstrated a drop in body weight to 70% of their original mass, or signs of severe morbidity were euthanized.

### Ethics statement

To prevent avoidable suffering, mice demonstrating a drop in body weight to 70% of their original mass, or signs of severe morbidity, were euthanized.

Mice were anesthetized with 0.1 ml/10 g body weight with ketamine HCL (9 mg/ml) and xylazine (1 mg/ml) by intraperitoneal injection. Alternatively, mice were anesthetized with CO_2_/O_2_. Mice were euthanized by first anesthetizing them with CO_2_/O_2_, followed by cervical dislocation.

This study was carried out in strict accordance with the recommendations in the Guide for the Care and Use of Laboratory Animals of the National Institutes of Health. Animal experiments were performed at Saint Louis University and approved by the Institutional Animal Care and Use Committee (#IACUC 2082). Additionally, these experiments were performed in accordance with mouse ethics outlined by the Canadian Council on Animal Care and the University of Alberta.

## Supporting Information

Figure S1 **ECTV encodes four ankyrin/F-box proteins.** (A) ECTV-encoded proteins, EVM002, EVM005, EVM154 and EVM165 contain a series of N-terminal ankyrin repeats in conjunction with a C-terminal F-box domain. EVM005(1-593) is a mutant that lacks the C-terminal F-box domain and has lost the ability to interact with the cellular SCF ubiquitin ligase [25]. (B) Cullin-1 serves as a scaffold protein for the cellular SCF ubiquitin ligase complex. Roc1 binds the C-terminus of cullin-1 and contains E3 ligase activity that recruits activated E2 enzymes that catalyze the formation of K48-linked polyubiquitin chains on substrate proteins. Substrate adaptor proteins utilize an F-box domain to interact with Skp1, the linker protein of the SCF ubiquitin ligase complex. Adaptor proteins recruit substrates through additional protein-protein interaction motifs.
(TIF)

Figure S2 **Construction and characterization of ECTV-Δ005.** (A) The Selectable and Excisable Marker system was used to delete the EVM005 gene from ECTV strain Moscow. We inserted a *yfp-gpt* fusion cassette flanked by loxP sites, and used the Cre recombinase to remove the marker following selection in order to construct a marker-free deletion virus. (B) PCR of viral genomes was used to demonstrate insertion and deletion of our *yfp-gpt* cassette, and purity of our virus stocks. (C) Multi-step growth curve analysis of ECTV compared to ECTV-Δ005, ECTV-005-rev and ECTV-005(1-593)-rev. BGMK cells were infected at a MOI of 0.05 and growth was monitored over 72 hours. The number of plaque forming units at each time point was measured by plaque assay. The average number of pfu at each time point was averaged from three independent experiments.
(TIF)

Figure S3 **Schematic for construction of ECTV-Δ002-005.** The Selectable and Excisable Marker System was used to delete four genes, EVM002, EVM003, EVM004, and EVM005 from the left end of the ECTV strain Moscow genome. (A) BGMK cells were infected with wild type ECTV and transfected with linearized pDGloxP-EVM002KO and YFP-GPT positive viruses were purified. (B) Excision of the *yfp-gpt* cassette was performed by infecting U20S-Cre cells with ECTV-Δ002-YFP-GPT to create ECTV-Δ002. (C) The *yfp-gpt* cassette was then inserted into the EVM005 locus by infecting BGMK cells with ECTV-Δ002 and transfecting linearized pDGloxP-EVM005KO and YFP-GPT positive viruses were purified. (D) Finally, we infected U20S-Cre cells with ECTV-Δ002/005-YFP-GPT to remove the *yfp-gpt* cassette. Cre recombination removed all DNA between the loxP site in the EVM002 locus and the loxP site introduced into the EVM005 locus, consisting of EVM002, EVM003, EVM004 and EVM005, to create ECTV-Δ002-005.
(TIF)

Figure S4 **PCR analysis of viral genomes to verify construction of ECTV-Δ002-005.** BGMK cells were infected with ECTV, ECTV-Δ002, ECTV-Δ002/005-YFP-GPT, and ECTV-Δ002-005 for 48 hours. Viral genomes were subjected to PCR analysis of the EVM002, EVM003, EVM004, and EVM005 loci as well as verifying Cre deletion from EVM002 to EVM005. The presence of PCR products near 500 bp in length represent excision of the *yfp-gpt* cassette by Cre recombination. Alternatively, PCR products of ∼1700 bp represent an intact *yfp-gpt* cassette, and PCR products larger than 2000 bp represent wild type sequences for EVM002 or EVM005. The presence of EVM004 is noted by the presence of a 822 bp PCR product. The presence of EVM003 is denoted by a 963 bp PCR product.
(TIF)

Figure S5 **Quantitative PCR analysis of cytokine induction following infection with ECTV or ECTV-Δ005.** HeLa cells were infected with ECTV or ECTV-Δ005 at a MOI of 5 for 12 hours. RNA from infected cells was harvested with Trizol and converted to cDNA by reverse transcription. The relative levels of TNFα, IL-1β and IL-6 were measured by real time PCR and normalized to GAPDH as well as uninfected cell transcript levels.
(TIF)

Figure S6 **Dose response curve to ECTV infection in C57BL/6 mice.** Groups of five female C57BL/6 mice were mock-infected or infected with ECTV, ECTV-Δ005, ECTV-005-rev, or ECTV-005(1-593)-rev via intranasal inoculation with 10-fold escalating doses between 10^2^ and 10^4^ pfu per mouse. Mice were monitored daily for body weight (A–C), day of death and mortality (D–F).
(TIF)

Figure S7 **Dose response curve to ECTV infection in A/NCR mice.** Groups of five female A/NCR mice were mock-infected or infected with ECTV, ECTV-Δ005, ECTV-005-rev or ECTV-005(1-593)-rev with 10-fold escalating doses between 10^1^ and 10^4^ pfu per mouse via footpad injection. Mice were monitored daily for body weight (A–D), day of death and mortality (E–H).
(TIF)

Figure S8 **EVM005 is expressed early during infection.** HeLa cells were infected with ECTV at a MOI of 5 in the presence or absence of the DNA replication inhibitor AraC. RNA was extracted from cells at the indicated time points post infection with Trizol and subjected to RT-PCR analysis. Primers specific for GAPDH as well as known early (EVM004) and late (EVM058) poxvirus genes served as controls.
(TIF)
