# Supplementary figures and images for: EVM005: An Ectromelia-Encoded Protein with Dual Roles in NF-κB Inhibition and Virulence
Source: PLoS Pathog. 2014 Aug 14;10(8):e1004326. doi: 10.1371/journal.ppat.1004326 (PMC4133408; doi:10.1371/journal.ppat.1004326)

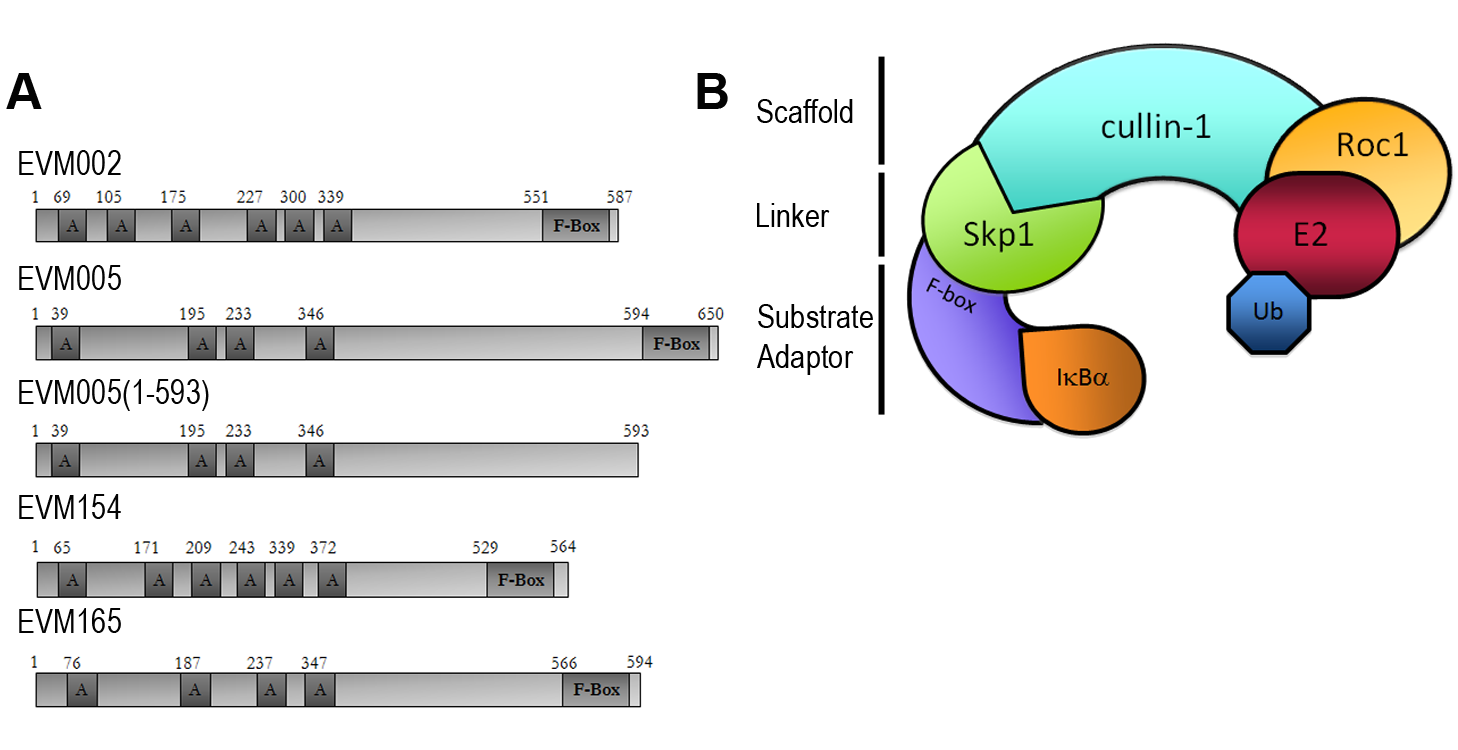

Supplement: Figure S1 — ECTV encodes four ankyrin/F-box proteins. (A) ECTV-encoded proteins, EVM002, EVM005, EVM154 and EVM165 contain a series of N-terminal ankyrin repeats in conjunction with a C-terminal F-box domain. EVM005(1-593) is a mutant that lacks the C-terminal F-box domain and has lost the ability to interact with the cellular SCF ubiquitin ligase [25]. (B) Cullin-1 serves as a scaffold protein for the cellular SCF ubiquitin ligase complex. Roc1 binds the C-terminus of cullin-1 and contains E3 ligase activity that recruits activated E2 enzymes that catalyze the formation of K48-linked polyubiquitin chains on substrate proteins. Substrate adaptor proteins utilize an F-box domain to interact with Skp1, the linker protein of the SCF ubiquitin ligase complex. Adaptor proteins recruit substrates through additional protein-protein interaction motifs. (TIF) [file ppat.1004326.s001.tif]

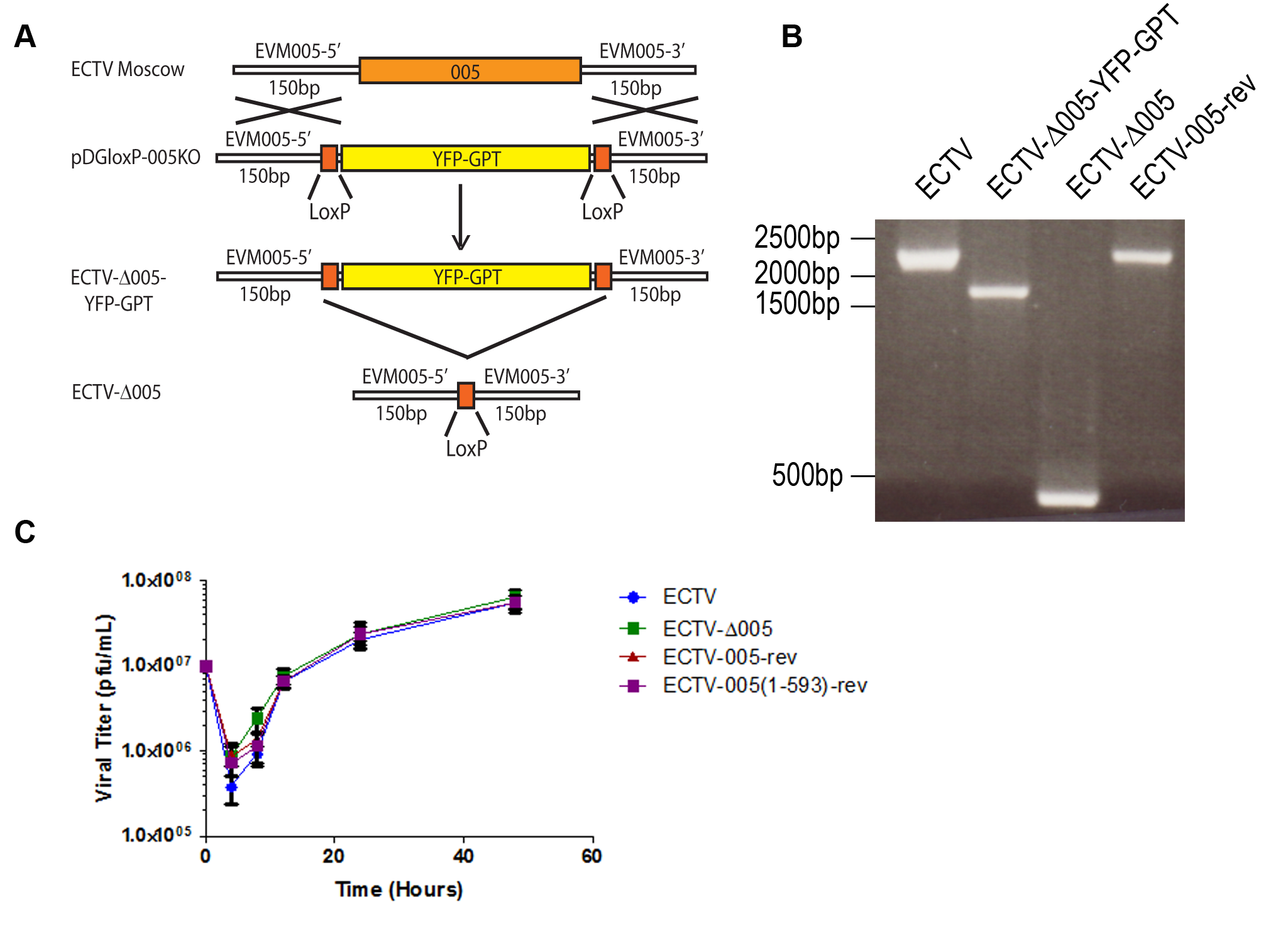

Supplement: Figure S2 — Construction and characterization of ECTV-Δ005. (A) The Selectable and Excisable Marker system was used to delete the EVM005 gene from ECTV strain Moscow. We inserted a yfp-gpt fusion cassette flanked by loxP sites, and used the Cre recombinase to remove the marker following selection in order to construct a marker-free deletion virus. (B) PCR of viral genomes was used to demonstrate insertion and deletion of our yfp-gpt cassette, and purity of our virus stocks. (C) Multi-step growth curve analysis of ECTV compared to ECTV-Δ005, ECTV-005-rev and ECTV-005(1-593)-rev. BGMK cells were infected at a MOI of 0.05 and growth was monitored over 72 hours. The number of plaque forming units at each time point was measured by plaque assay. The average number of pfu at each time point was averaged from three independent experiments. (TIF) [file ppat.1004326.s002.tif]

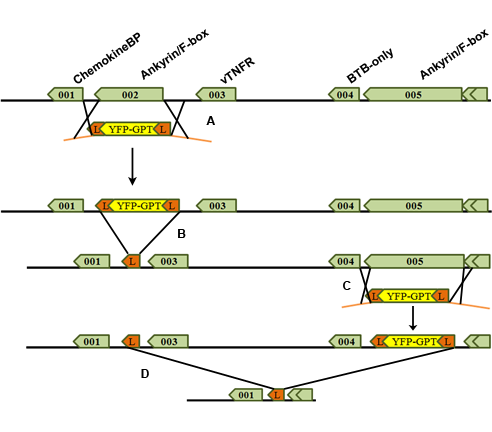

Supplement: Figure S3 — Schematic for construction of ECTV-Δ002-005. The Selectable and Excisable Marker System was used to delete four genes, EVM002, EVM003, EVM004, and EVM005 from the left end of the ECTV strain Moscow genome. (A) BGMK cells were infected with wild type ECTV and transfected with linearized pDGloxP-EVM002KO and YFP-GPT positive viruses were purified. (B) Excision of the yfp-gpt cassette was performed by infecting U20S-Cre cells with ECTV-Δ002-YFP-GPT to create ECTV-Δ002. (C) The yfp-gpt cassette was then inserted into the EVM005 locus by infecting BGMK cells with ECTV-Δ002 and transfecting linearized pDGloxP-EVM005KO and YFP-GPT positive viruses were purified. (D) Finally, we infected U20S-Cre cells with ECTV-Δ002/005-YFP-GPT to remove the yfp-gpt cassette. Cre recombination removed all DNA between the loxP site in the EVM002 locus and the loxP site introduced into the EVM005 locus, consisting of EVM002, EVM003, EVM004 and EVM005, to create ECTV-Δ002-005. (TIF) [file ppat.1004326.s003.tif]

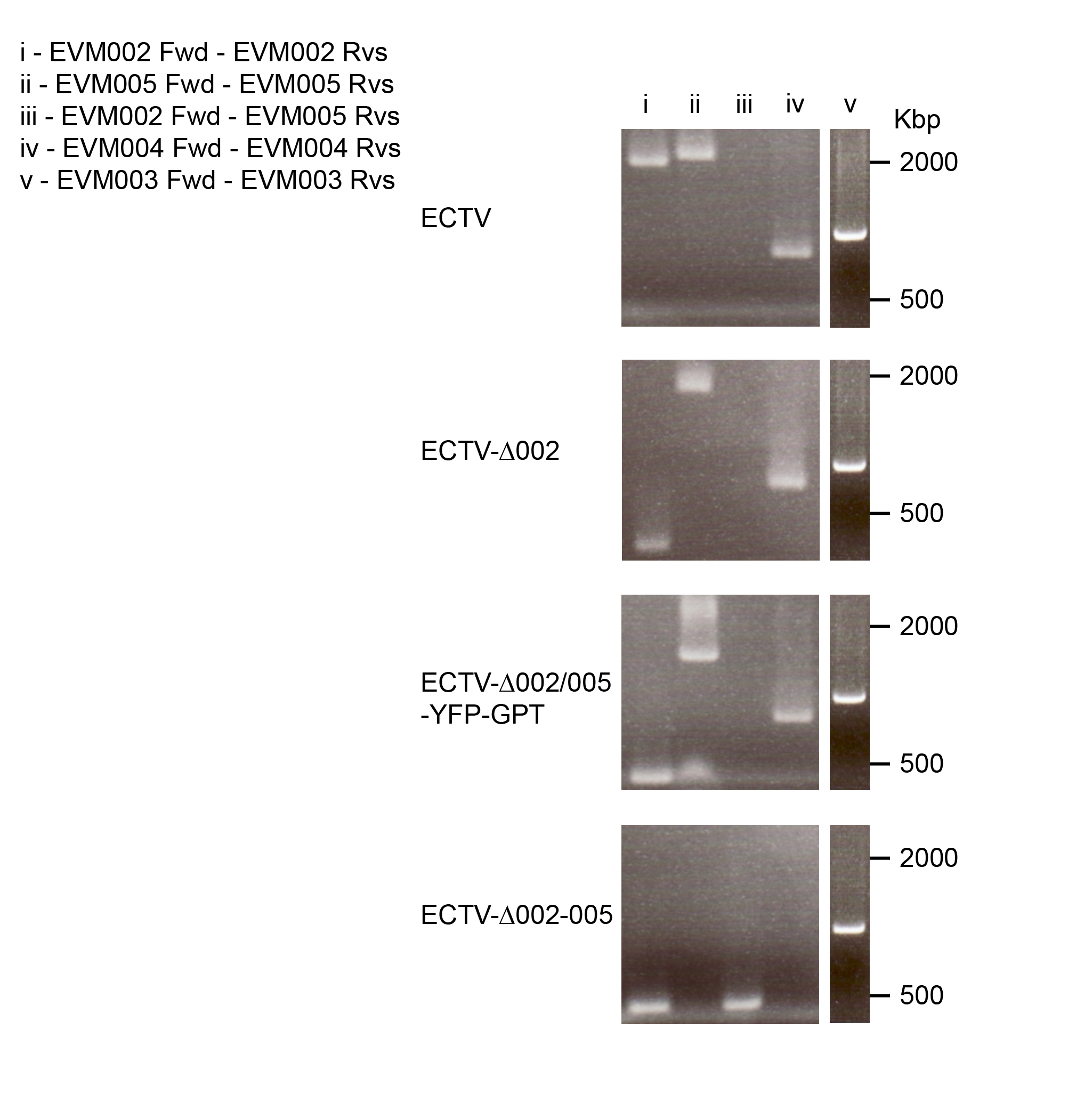

Supplement: Figure S4 — PCR analysis of viral genomes to verify construction of ECTV-Δ002-005. BGMK cells were infected with ECTV, ECTV-Δ002, ECTV-Δ002/005-YFP-GPT, and ECTV-Δ002-005 for 48 hours. Viral genomes were subjected to PCR analysis of the EVM002, EVM003, EVM004, and EVM005 loci as well as verifying Cre deletion from EVM002 to EVM005. The presence of PCR products near 500 bp in length represent excision of the yfp-gpt cassette by Cre recombination. Alternatively, PCR products of ∼1700 bp represent an intact yfp-gpt cassette, and PCR products larger than 2000 bp represent wild type sequences for EVM002 or EVM005. The presence of EVM004 is noted by the presence of a 822 bp PCR product. The presence of EVM003 is denoted by a 963 bp PCR product. (TIF) [file ppat.1004326.s004.tif]

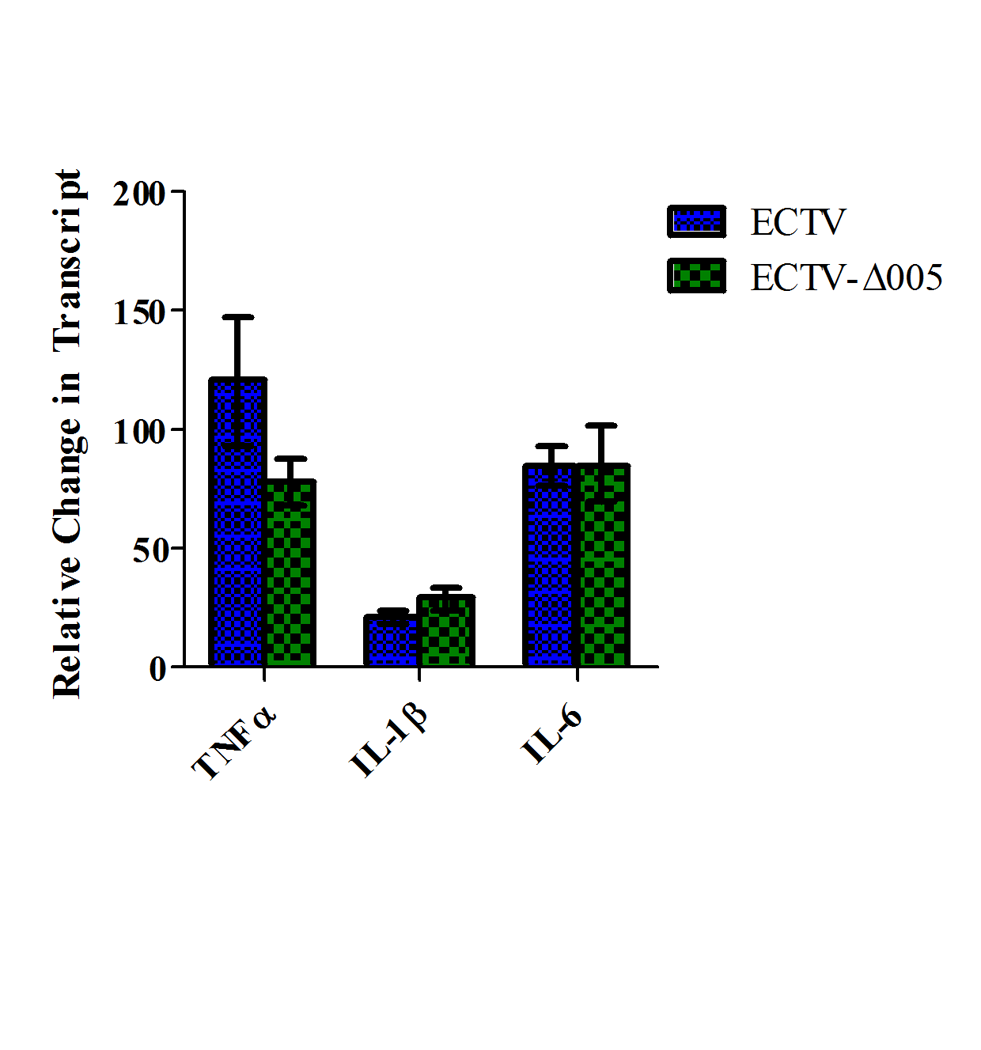

Supplement: Figure S5 — Quantitative PCR analysis of cytokine induction following infection with ECTV or ECTV-Δ005. HeLa cells were infected with ECTV or ECTV-Δ005 at a MOI of 5 for 12 hours. RNA from infected cells was harvested with Trizol and converted to cDNA by reverse transcription. The relative levels of TNFα, IL-1β and IL-6 were measured by real time PCR and normalized to GAPDH as well as uninfected cell transcript levels. (TIF) [file ppat.1004326.s005.tif]

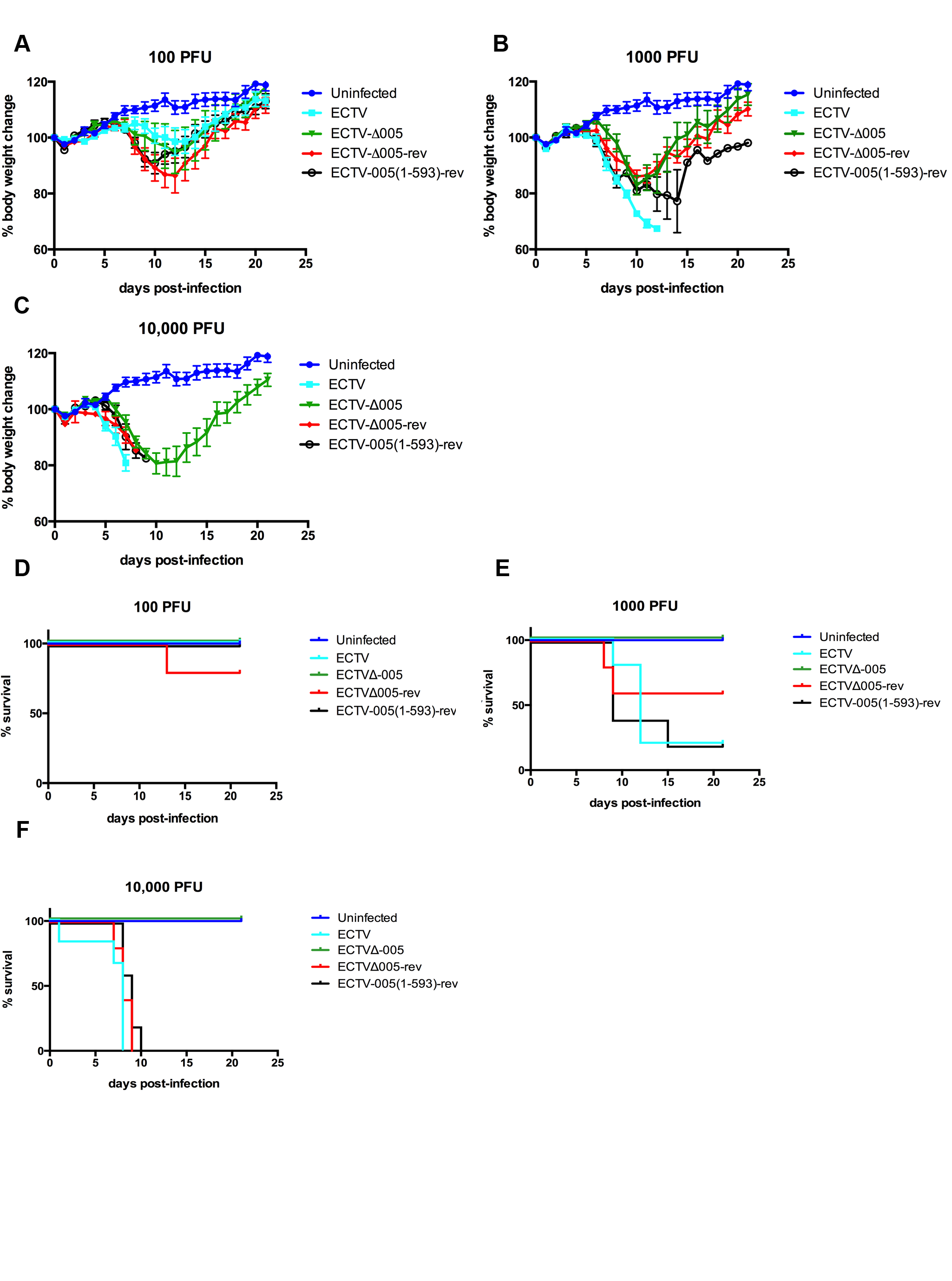

Supplement: Figure S6 — Dose response curve to ECTV infection in C57BL/6 mice. Groups of five female C57BL/6 mice were mock-infected or infected with ECTV, ECTV-Δ005, ECTV-005-rev, or ECTV-005(1-593)-rev via intranasal inoculation with 10-fold escalating doses between 102 and 104 pfu per mouse. Mice were monitored daily for body weight (A–C), day of death and mortality (D–F). (TIF) [file ppat.1004326.s006.tif]

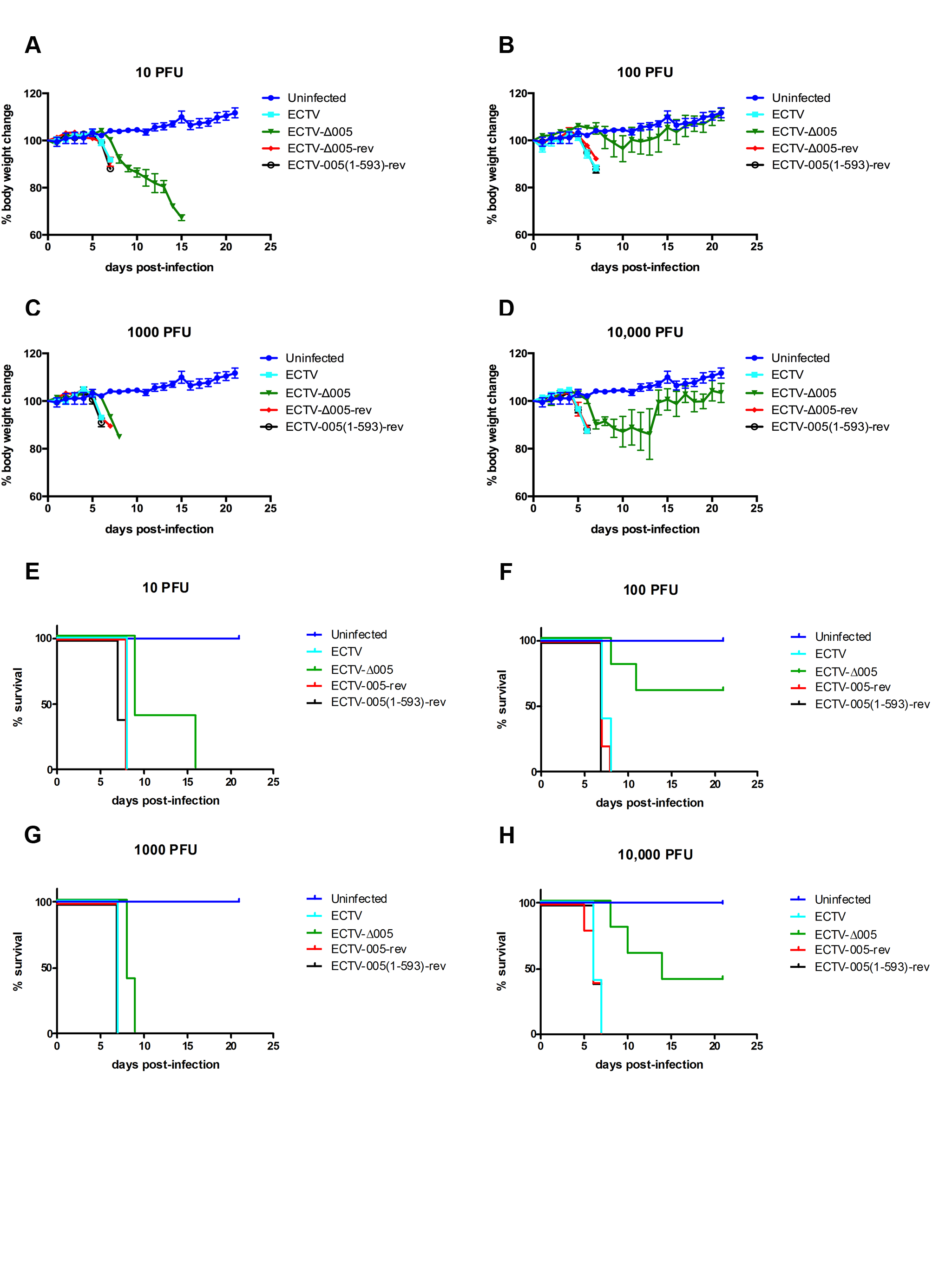

Supplement: Figure S7 — Dose response curve to ECTV infection in A/NCR mice. Groups of five female A/NCR mice were mock-infected or infected with ECTV, ECTV-Δ005, ECTV-005-rev or ECTV-005(1-593)-rev with 10-fold escalating doses between 101 and 104 pfu per mouse via footpad injection. Mice were monitored daily for body weight (A–D), day of death and mortality (E–H). (TIF) [file ppat.1004326.s007.tif]

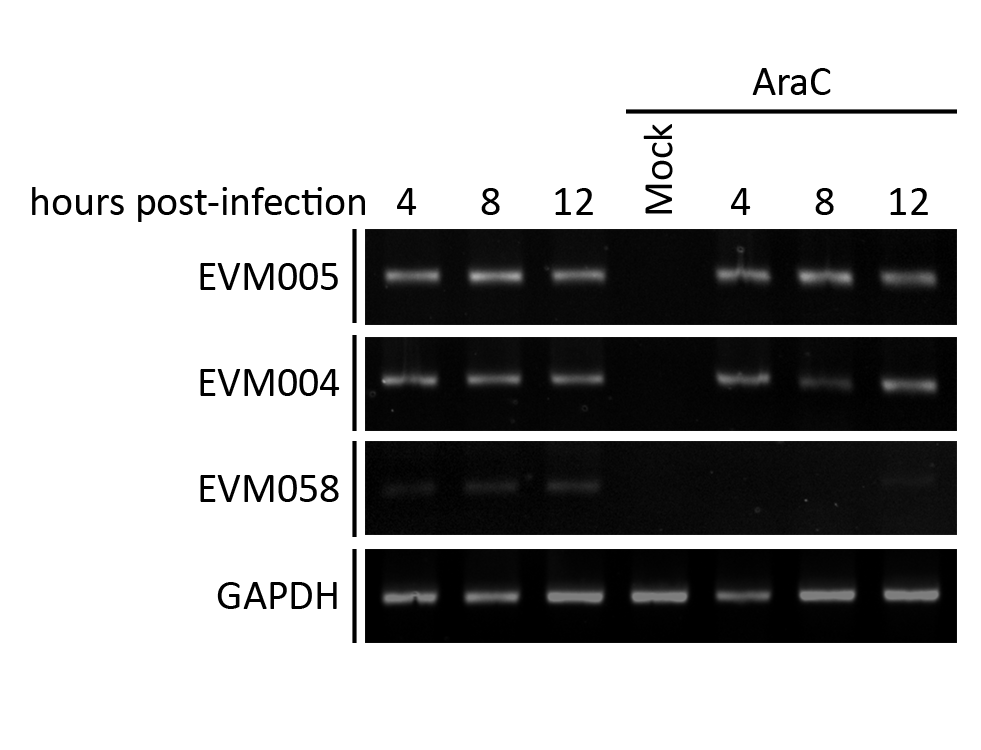

Supplement: Figure S8 — EVM005 is expressed early during infection. HeLa cells were infected with ECTV at a MOI of 5 in the presence or absence of the DNA replication inhibitor AraC. RNA was extracted from cells at the indicated time points post infection with Trizol and subjected to RT-PCR analysis. Primers specific for GAPDH as well as known early (EVM004) and late (EVM058) poxvirus genes served as controls. (TIF) [file ppat.1004326.s008.tif]
